# Supplementary material for: Can you hear me? Playback experiment highlights detection range differences between commonly used PAM devices: C-POD, F-POD and SoundTrap
Source: PLoS One. 2025 Apr 9;20(4):e0320925. doi: 10.1371/journal.pone.0320925 (PMC11981142; doi:10.1371/journal.pone.0320925)
Supplement: S1 File — (DOCX) [file pone.0320925.s001.docx]

S1 File- Criteria for identifying positive detections of playbacks based on spectrograms

SoundTrap wav files were imported to Audacity for the visual inspection of spectrograms during the playback period. Prior to the inspection of spectrograms the SoundTrap wav files were cropped so that one wav file exists per playback station. In order to determine whether a playback click was detected or not detected the spectrograms were assessed following several key steps (see below).

1. The full spectrogram per playback station was inspected noting any evidence of harbour porpoise playback clicks in the high frequency band and lower frequency ambient or boat noise (Figure 1).


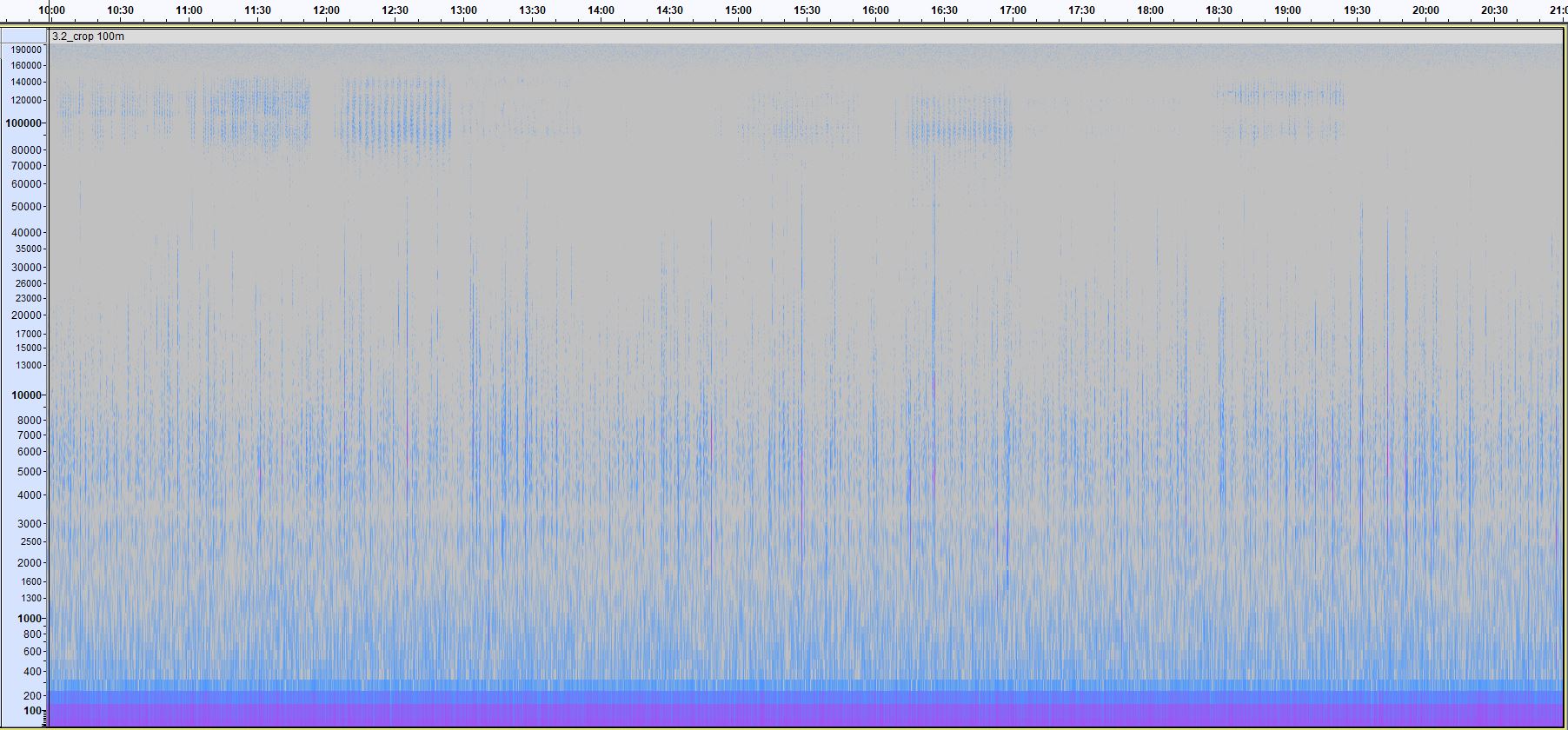


S1 Fig. Full spectrogram from SoundTrap at 100m from the playback station.

1. The higher frequency band was more closely examined (~100kHz). In this case, there are clear repeated sequences visible (Figure 2).


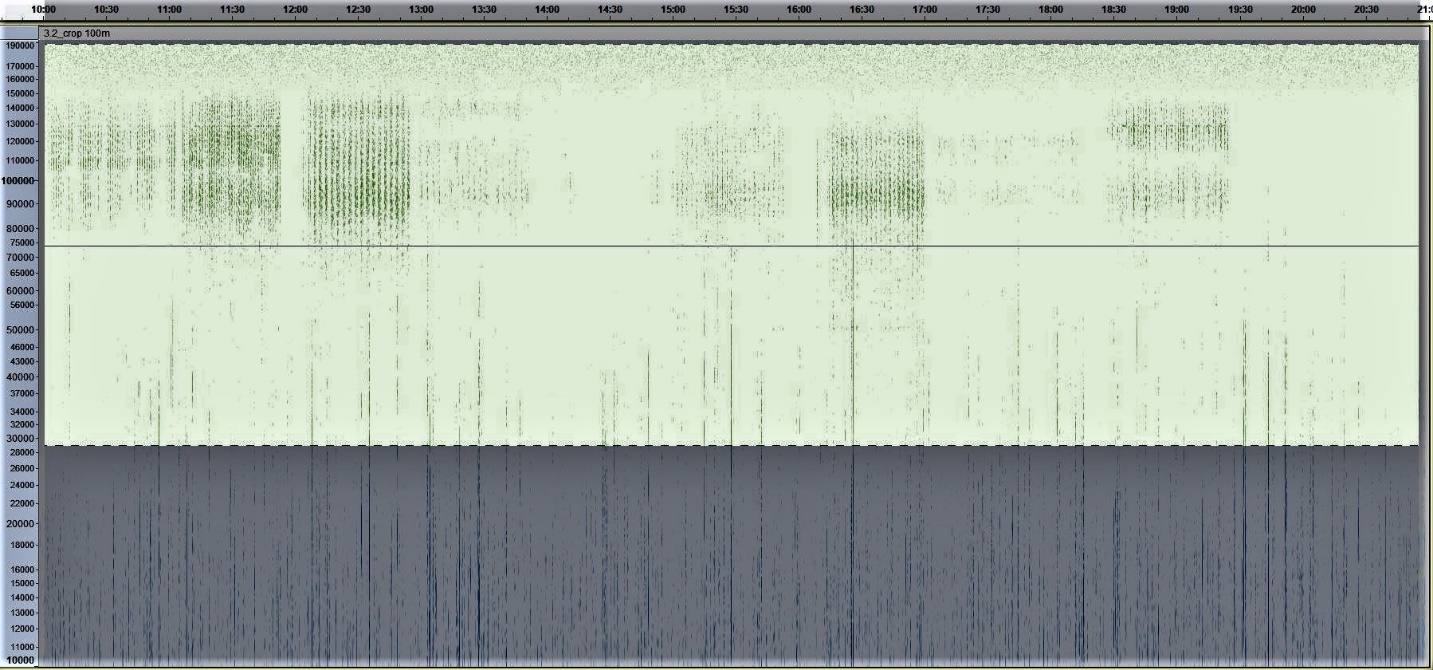


S2 Fig. Spectrogram from SoundTrap at 100m zoomed to the high frequency band

1. This frequency band was exported from Audacity, zoomed in further for more precise inspection and annotated with time stamps, and marking the timing of the individual playback clicks according to data recording forms (Figure 3). Detection or no detection was then recorded if evidence of playback within these marked areas. See below, detections for all playbacks except rec 5-50dB.


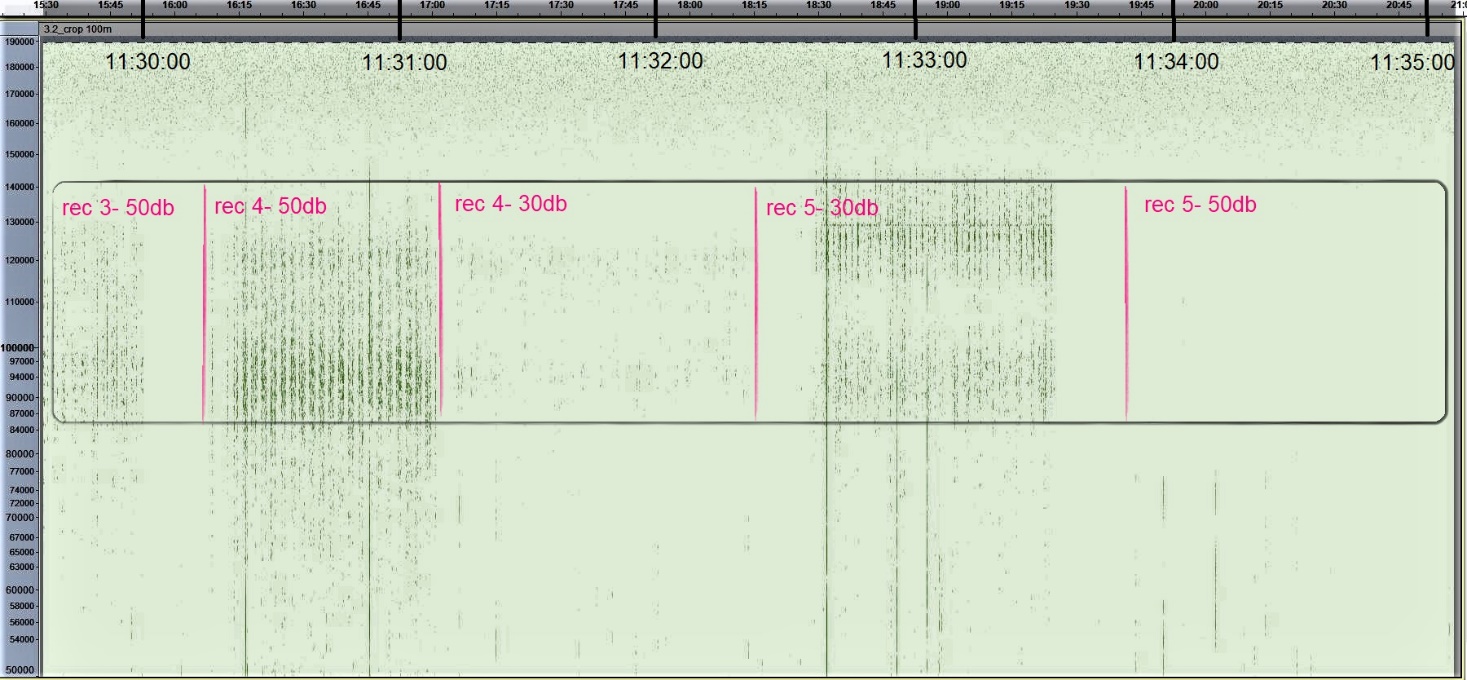


S3 Fig. Spectrogram from SoundTrap at 100m, zoomed and annotated with timestamps and specific playback durations.

1. A control period, during which no playbacks were transmitted, was also analysed to confirm that recorded detections for each playback were accurate and truly associated with the playbacks. As shown in Figure 4, some noise spikes were recorded at high frequencies during the control period. Therefore, to be recognised as a valid detection, a playback signal must be consistently recorded within the high-frequency band (as highlighted in Figure 3) throughout the entire minute of the playback’s transmission. NB: Some of the playbacks can be detected outside of this high frequency band due to low frequency artefacts in the recordings, however only the high frequency band was examined for positive detections.


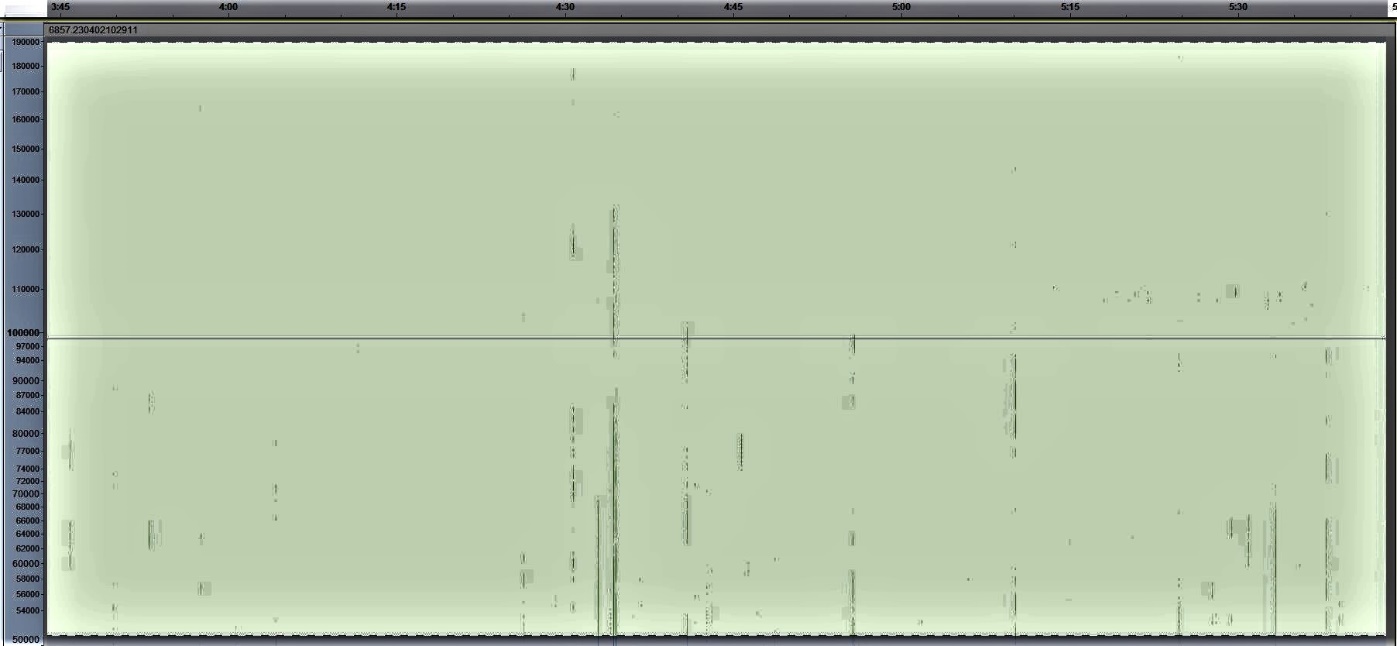


S4 Fig. Spectrogram from SoundTrap prior to the commencing of the playback experiment, zoomed to the high frequency band to examine region for a control.
