# Supplementary material for: Can you hear me? Playback experiment highlights detection range differences between commonly used PAM devices: C-POD, F-POD and SoundTrap
Source: PLoS One. 2025 Apr 9;20(4):e0320925. doi: 10.1371/journal.pone.0320925 (PMC11981142; doi:10.1371/journal.pone.0320925)
Supplement: S2 File — Model parameters and AIC values for model iterations used in model selection. Stepwise model selection was performed where non-significant interactions were dropped from the model (starting with the least significant) and model validation was repeated. Models were compared using AIC to choose the best and final model. Chosen model highlighted in bold red for each device. (DOCX) [file pone.0320925.s002.docx]

S2 File- AIC values used in the model selection process

|  | C-POD |  | F-POD |  | SoundTrap |
| --- | --- | --- | --- | --- | --- |
| Parameters | AIC | Parameters | AIC | Parameters | AIC |
| Distance + depth + transect + source level + tide | 63.92877 | Distance + depth + transect + source level + tide | 94.14105 | Distance + depth + transect + source level + tide | 118.8639 |
| Distance + depth + transect + source level | 61.93182 | Distance + depth + transect + source level | 92.1539 | Distance + depth + transect + source level | **116.9709** |
| Distance + transect + source level | **60.11972** | Distance + depth + source level | 94.74441 | Distance + depth + source level | 118.7112 |
| Distance + transect | 69.35053 | Distance + depth | **90.83254** | Distance + source level | 118.8099 |
|  |  | distance | 93.07361 | distance | 135.5195 |

**S5 Table.** Model parameters and AIC values for model iterations used in model selection. Stepwise model selection was performed where non-significant interactions were dropped from the model (starting with the least significant) and model validation was repeated. Models were compared using AIC to choose the best and final model. Chosen model highlighted in bold red for each device.
