# Supplementary material for: Can you hear me? Playback experiment highlights detection range differences between commonly used PAM devices: C-POD, F-POD and SoundTrap
Source: PLoS One. 2025 Apr 9;20(4):e0320925. doi: 10.1371/journal.pone.0320925 (PMC11981142; doi:10.1371/journal.pone.0320925)
Supplement: Attachment — Submitted filename: PONE-D-24-43959_reviewer.pdf [file pone.0320925.s003.pdf]

PLOS ONE

Can you hear me? Playback experiment highlights porpoise detection range differences between commonly used PAM devices: C-POD, F-POD, and SoundTrap.

--Manuscript Draft--

**Manuscript Number:**	PONE-D-24-43959
**Article Type:**	Research Article
**Full Title:**	Can you hear me? Playback experiment highlights porpoise detection range differences between commonly used PAM devices: C-POD, F-POD, and SoundTrap.
**Short Title:**	Playback experiment highlights detection range differences between PAM devices
**Corresponding Author:**	Nicole Rose Eileen Todd<br>University College Cork Environmental Research Institute<br>Cork, IRELAND
**Keywords:**	detection function; passive acoustic monitoring; Phocoena phocoena; C-POD; F-POD; SoundTrap
**Abstract:**	Passive acoustic monitoring (PAM) is a valuable tool for monitoring acoustically active small cetaceans such as the harbour porpoise (Phocoena phocoena), with a range of devices commonly used across studies. However, to ensure comparability of findings, there is a need to compare the ability of devices to detect vocalisations. Using a playback approach, we determine detection probability and effective detection radius/area (EDR/EDA) for co-deployed C-POD (Cetacean POrpoise Detectors), F-POD (Full waveform capture POD) and SoundTrap acoustic monitoring devices. We conducted playbacks of harbour porpoise vocalisations across two transects at a range of distances from moored devices, while accounting for a range of variables likely to influence the detection probability of playbacks. Distance from the devices influenced the detection probability across all devices, and a significant difference between transects was also found for the C-POD, possibly due to different ambient noise conditions. The maximum detection distance of the playbacks for the SoundTrap and the F-POD was between 400 - 500m, and EDR was estimated at 297m (EDA 0.276 km2) and 241m (EDA 0.181 km2), respectively. The maximum detection distance for the C-POD was lower, at 300 - 400m, and an EDR of 220m (EDA 0.153 km2). A lower EDR was calculated for harbour porpoise buzzes compared to clicks across devices, due to lower source level of buzzes, suggesting that time spent foraging may be underestimated in PAM studies. The results highlight how detection ranges may differ across commonly used PAM devices, affecting comparability of detection rates across studies. EDR/EDA is an important prerequisite for PAM-derived density and abundance estimates. As such, understanding how devices differ is essential for comparing studies and appropriate planning of long-term acoustic monitoring projects, particularly where estimates of abundance are a key goal.
**Order of Authors:**	Nicole Rose Eileen Todd
	Ailbhe Sarah Kavanagh
	Mark Jessopp
	Willem Verboom
	Emer Rogan
**Additional Information:**	
**Question**	**Response**
**Financial Disclosure**<br><br>Enter a financial disclosure statement that describes the sources of funding for the work included in this submission. Review the submission guidelines for detailed	Yes

Powered by Editorial Manager® and ProduXion Manager® from Aries Systems Corporation

requirements. View published research articles from *PLOS ONE* for specific examples.<br><br>This statement is required for submission and **will appear in the published article** if the submission is accepted. Please make sure it is accurate.<br><br>**Funded studies**<br>Enter a statement with the following details:<br>• Initials of the authors who received each award<br>• Grant numbers awarded to each author<br>• The full name of each funder<br>• URL of each funder website<br>• Did the sponsors or funders play any role in the study design, data collection and analysis, decision to publish, or preparation of the manuscript?<br><br>Did you receive funding for this work?	
Please add funding details.<br>as follow-up to "**Financial Disclosure**<br><br>Enter a financial disclosure statement that describes the sources of funding for the work included in this submission. Review the submission guidelines for detailed requirements. View published research articles from *PLOS ONE* for specific examples.<br><br>This statement is required for submission and **will appear in the published article** if the submission is accepted. Please make sure it is accurate.	This work was funded by the Irish Research Council Government of Ireland Postgraduate Scholarship Scheme (Project ID: GOIPG/2019/2173).

**Funded studies**<br>Enter a statement with the following details:<br>• Initials of the authors who received each award<br>• Grant numbers awarded to each author<br>• The full name of each funder<br>• URL of each funder website<br>• Did the sponsors or funders play any role in the study design, data collection and analysis, decision to publish, or preparation of the manuscript?<br><br>Did you receive funding for this work?"	
Please select the country of your main research funder (please select carefully as in some cases this is used in fee calculation).<br>as follow-up to "**Financial Disclosure**<br><br>Enter a financial disclosure statement that describes the sources of funding for the work included in this submission. Review the submission guidelines for detailed requirements. View published research articles from *PLOS ONE* for specific examples.<br><br>This statement is required for submission and **will appear in the published article** if the submission is accepted. Please make sure it is accurate.<br><br>**Funded studies**<br>Enter a statement with the following details:<br>• Initials of the authors who received each award<br>• Grant numbers awarded to each author<br>• The full name of each funder<br>• URL of each funder website<br>• Did the sponsors or funders play any role in the study design, data collection and analysis, decision to publish, or preparation of the manuscript?<br><br>Did you receive funding for this work?"	IRELAND - IE
**Competing Interests**	The authors have declared that no competing interests exist.

Use the instructions below to enter a competing interest statement for this submission. On behalf of all authors, disclose any competing interests that could be perceived to bias this work—acknowledging all financial support and any other relevant financial or non-financial competing interests.<br><br>This statement is required for submission and **will appear in the published article** if the submission is accepted. Please make sure it is accurate and that any funding sources listed in your Funding Information later in the submission form are also declared in your Financial Disclosure statement.<br><br>View published research articles from *PLOS ONE* for specific examples.<br><br>**NO authors have competing interests**<br><br>Enter: *The authors have declared that no competing interests exist.*<br><br>**Authors with competing interests**<br><br>Enter competing interest details beginning with this statement:<br><br>*I have read the journal's policy and the authors of this manuscript have the following competing interests: [insert competing interests here]*<br><br>* typeset	
**Ethics Statement**<br><br>Enter an ethics statement for this submission. This statement is required if the study involved:<br><br>• Human participants<br>• Human specimens or tissue<br>• Vertebrate animals or cephalopods<br>• Vertebrate embryos or tissues	N/A

Powered by Editorial Manager® and ProduXion Manager® from Aries Systems Corporation

• Field research<br><br>Write "N/A" if the submission does not require an ethics statement.<br><br>General guidance is provided below. Consult the submission guidelines for detailed instructions. **Make sure that all information entered here is included in the Methods section of the manuscript.**	

Powered by Editorial Manager® and ProduXion Manager® from Aries Systems Corporation

**Format for specific study types**<br><br>**Human Subject Research (involving human participants and/or tissue)**<br>• Give the name of the institutional review board or ethics committee that approved the study<br>• Include the approval number and/or a statement indicating approval of this research<br>• Indicate the form of consent obtained (written/oral) or the reason that consent was not obtained (e.g. the data were analyzed anonymously)<br><br>**Animal Research (involving vertebrate animals, embryos or tissues)**<br>• Provide the name of the Institutional Animal Care and Use Committee (IACUC) or other relevant ethics board that reviewed the study protocol, and indicate whether they approved this research or granted a formal waiver of ethical approval<br>• Include an approval number if one was obtained<br>• If the study involved *non-human primates*, add *additional details* about animal welfare and steps taken to ameliorate suffering<br>• If anesthesia, euthanasia, or any kind of animal sacrifice is part of the study, include briefly which substances and/or methods were applied<br><br>**Field Research**<br><br>Include the following details if this study involves the collection of plant, animal, or other materials from a natural setting:<br>• Field permit number<br>• Name of the institution or relevant body that granted permission	
**Data Availability**<br><br>Authors are required to make all data underlying the findings described fully available, without restriction, and from the time of publication. PLOS allows rare exceptions to address legal and ethical concerns. See the PLOS Data Policy and FAQ for detailed information.	Yes - all data are fully available without restriction

A Data Availability Statement describing where the data can be found is required at submission. Your answers to this question constitute the Data Availability Statement and **will be published in the article**, if accepted.<br><br>**Important:** Stating 'data available on request from the author' is not sufficient. If your data are only available upon request, select 'No' for the first question and explain your exceptional situation in the text box.<br><br>Do the authors confirm that all data underlying the findings described in their manuscript are fully available without restriction?	
**Describe where the data may be found in full sentences. If you are copying our sample text, replace any instances of XXX with the appropriate details.**<br><br>• If the data are **held or will be held in a public repository**, include URLs, accession numbers or DOIs. If this information will only be available after acceptance, indicate this by ticking the box below. For example: *All XXX files are available from the XXX database (accession number(s) XXX, XXX.*).<br>• If the data are all contained **within the manuscript and/or Supporting Information files**, enter the following: *All relevant data are within the manuscript and its Supporting Information files.*<br>• If neither of these applies but you are able to provide **details of access elsewhere**, with or without limitations, please do so. For example:<br><br>*Data cannot be shared publicly because of [XXX]. Data are available from the XXX Institutional Data Access / Ethics Committee (contact via XXX) for researchers who meet the criteria for access to confidential data.*<br><br>*The data underlying the results presented in the study are available from (include the name of the third party*	All processed data and R code files to reproduce the results given in this paper are available in the following repository https://github.com/Ntodd95/playbacks

and contact information or URL).<br>• This text is appropriate if the data are owned by a third party and authors do not have permission to share the data.<br><br>* typeset	
Additional data availability information:	Tick here if the URLs/accession numbers/DOIs will be available only after acceptance of the manuscript for publication so that we can ensure their inclusion before publication.

Powered by Editorial Manager® and ProduXion Manager® from Aries Systems Corporation

manuscript Click here to access/download;Manuscript;Can you hear me manuscript_.docx

Full title: Can you hear me? Playback experiment highlights porpoise detection range differences between commonly used PAM devices: C-POD, F-POD, and SoundTrap

Short title: Playback experiment highlights detection range differences between PAM devices

Authors names: Nicole R. E. Todd [1,2]***,** Ailbhe S. Kavanagh[3], Mark J. Jessopp[1,2], Willem Verboom[4], Emer Rogan[2]

1 MaREI Centre, Environmental Research Institute, University College Cork
2 School of Biological, Earth & Environmental Sciences (BEES), University College Cork
3 Fisheries Ecosystems Advisory Services (FEAS), Marine Institute, Oranmore, Co. Galway, Ireland
4 JunoBioacoustics, Winkel, The Netherlands
* Corresponding author
Email: nicole.todd@ucc.ie

Author contributions: This study was conceptualized and methodology defined by N.R.E.T/A.S.K/M.J.J/E.R. Data was curated by N.R.E.T/W.V. Formal analysis and investigation was undertaken by N.R.E.T. Original draft preparation prepared by N.R.E.T. All authors contributed to review and editing of manuscript. Funding acquisition N.R.E.T/A.S.K/M.J.J/E.R.

Abstract

Passive acoustic monitoring (PAM) is a valuable tool for monitoring acoustically active small cetaceans such as the harbour porpoise (*Phocoena phocoena*), with a range of devices commonly used across studies. However, to ensure comparability of findings, there is a need to compare the ability of devices to detect vocalisations. Using a playback approach, we determine detection probability and effective detection radius/area (EDR/EDA) for co-deployed C-POD (Cetacean POrpoise Detectors), F-POD (Full waveform capture POD) and SoundTrap acoustic monitoring devices. We conducted playbacks of harbour porpoise vocalisations across two transects at a range of distances from moored devices, while accounting for a range of variables likely to influence the detection probability of playbacks. Distance from the devices influenced the detection probability across all devices, and a significant difference between transects was also found for the C-POD, possibly due to different ambient noise conditions. The maximum detection distance of the playbacks for the SoundTrap and the F-POD was between 400 - 500m, and EDR was estimated at 297m (EDA 0.276 km^2) and 241m (EDA 0.181 km^2), respectively. The maximum detection distance for the C-POD was lower, at 300 - 400m, and an EDR of 220m (EDA 0.153 km^2). A lower EDR was calculated for harbour porpoise buzzes compared to clicks across devices, due to lower source level of buzzes, suggesting that time spent foraging may be underestimated in PAM studies. The results highlight how detection ranges may differ across commonly used PAM devices, affecting comparability of detection rates across studies. EDR/EDA is an important prerequisite for PAM-derived density and abundance estimates. As such, understanding how devices differ is essential for comparing studies and appropriate planning of long-term acoustic monitoring projects, particularly where estimates of abundance are a key goal.

Introduction

For sound-producing animals, passive acoustic monitoring (PAM) is an efficient, non-invasive technique to sample populations and make inferences about species distribution and occurrence over space and time. Passive acoustic methods are well suited for monitoring cetacean species that are difficult to monitor visually, such as deep diving species e.g., beaked whales (Barlow *et al.,* 2021a), or smaller cetaceans with cryptic surfacing behaviour, including

several species of porpoise (Davis *et al.,* 2022). The use of PAM-derived data to estimate animal density and abundance is becoming more widely used and has been reviewed for both terrestrial and marine settings (Blumstein *et al.,* 2011; Marques *et al.,* 2013). The application of such studies has been successful in the marine environment, in particular for cetaceans, where acoustic methods have been used to estimate the density of *inter alia* fin whales (*Balaenoptera physalus*) (Harris *et al.,* 2018), deep diving beaked whales (Blainville's (*Mesoplodon densirostris*) (Marques *et al.,* 2009), Cuvier's (*Ziphius cavirostris*) (Zimmer *et al.,* 2008; Barlow *et al.,* 2021b) and harbour porpoise (Amundin *et al.,* 2022).

Various PAM technologies have been used for studying cetaceans, including towed or drifting hydrophone arrays (Sveegaard *et al.,* 2011; Barlow *et al.,* 2018; Malinka *et al.,* 2020), fibre optic cables (Bouffaut *et al.,* 2022), ocean gliders (Cauchy *et al.,* 2023) and bottom-mounted static devices (e.g. Van Parijs *et al.,* 2009). Bottom-mounted, static PAM devices can be loosely categorised as either those that record full bandwidth raw acoustic data (e.g. SoundTrap, Ocean Instruments; Autonomous Multichannel Acoustic Recorders (AMARs), JASCO Applied Sciences; and HydroMoth, Open Acoustic Devices) or as click detectors with onboard detection algorithms that store acoustic information about the detected sounds (e.g. Cetacean POrpoise Detectors (C-PODs) and their successor, the Full waveform capture PODS (F-PODs), Chelonia Ltd.). Click detectors record information such as date and time, peak frequency, and amplitude, but they do not retain raw acoustic files for processing. Static PAM devices provide high-resolution data over long monitoring periods at discrete locations and have been widely used to monitor small coastal cetaceans such as the harbour porpoise (e.g., Carlström, 2005; Schaffeld *et al.,* 2016; Zein *et al.,* 2019; Todd *et al.,* 2020, 2022). To better interpret the resultant long-term PAM datasets, and ensure appropriate coverage of a survey area, it is important to consider the detection ranges of the devices used for the species of interest.

The detection range of a static PAM device is often referred to as the effective detection radius (EDR). This is a key prerequisite for density and abundance estimation using PAM (Marques *et al.,* 2013). The EDR is defined as the distance at which the number of missed detections near the detector is equal to the number of recorded detections at greater distances, within a maximum truncation distance (Barlow *et al.,* 2021b). Subsequently, EDR can be converted to an effective detection area (EDA), representing the spatial area monitored, that can be used for calculations of density and abundance. To obtain accurate detection distances, and estimate EDR/EDA for static PAM devices, studies have used simultaneous visual and acoustic surveys (Kyhn *et al.,* 2012; Jacobson *et al.,* 2017a), as well as playbacks of artificial cetacean clicks or recordings of vocalisations (Nuuttila *et al.,* 2018; Amundin *et al.,* 2022). For monitoring harbour porpoises, EDR has been estimated for T-PODs (the predecessor of the C-POD; Kyhn *et al.,* 2012), as well as more recently for F-POD and SoundTrap (Cosentino et al., 2023) and EDA has been estimated for C-PODs (Nuuttila *et al.,* 2018; Amundin *et al.,* 2022). However, to the best of our knowledge, no studies have compared the detection range of these commonly used PAM devices within the same soundscape setting. Many variables may influence the propagation of sound through water, including water temperature, depth, ambient noise, salinity, and seabed substrate (through transmission loss/ absorption by the sediment or through obstruction from physical objects) (Au & Hastings, 2008; Zimmer, 2011).

Comparative studies have shown that SoundTraps can detect as much as 50% more harbour porpoise detections when deployed alongside a C-POD (Sarnocinska *et al.,* 2016; Jacobson *et al.,* 2017b). Furthermore, co-deployed C-POD and F-PODs showed that the F-POD consistently logged significantly more harbour porpoise detections and detected nuanced temporal patterns in foraging behaviour that the C-POD failed to detect (Todd *et al.,* 2023). This poses the questions of (i) how comparable static PAM devices are in terms of their detection ranges, and (ii) what factors influence the detection rates across devices. This study aims to address this knowledge gap by (i) estimating the detection ranges (as EDR & EDA) of three commonly used static PAM devices within the same soundscape, and (ii) assessing the effect harbour porpoise echolocation behaviour/vocalisation type may have on these estimated detection ranges.

Materials and Methods

Deployment site and conditions

The deployment site is a relatively sheltered site on the southwest coast of Sherkin Island (SW Ireland, 51°27′40.7″ N, 9°26′24.7″ W), situated within Roaringwater Bay and Islands Special Area of Conservation (SAC). This SAC is designated for and supports harbour porpoise throughout the year (National Parks & Wildlife Service, 2014). Three static PAM devices were deployed, attached to an anchored mooring line in approximately 18m water depth, in an area with a predominantly sandy seafloor. A C-POD and an F-POD were co-deployed side by side in a custom-built acetal plastic frame secured to a mooring line at a depth of approximately 13m, with a SoundTrap600HF (Ocean Instruments, New Zealand) deployed immediately above this on the same line. The SoundTrap was configured on high gain, with a high pass filter and a sampling frequency of 384 kHz.

Playbacks of harbour porpoise vocalisations were conducted from a rigid inflatable boat, drifting with engines off to avoid engine noise in a sea state of Beaufort 2 (wind speed of 4 - 6kts, small wavelets with no whitecaps). Before commencing playbacks, a 30-minute visual survey of the area was conducted for the presence of any cetacean species, and playbacks were only carried out if no animals were sighted within a visible radius of approximately 5 km from the vessel and PAM mooring. Visual observations were also conducted during the transmission of the playbacks to ensure that no cetacean species knowingly entered the area during the playback experiment.

Playback recordings

Recorded vocalisations from captive harbour porpoises were used to investigate the detection ranges of C-POD, F-POD and SoundTrap in the field. Echolocation clicks were recorded from two harbour porpoises at Harderwijk Marine Mammal Park, Netherlands,

housed in an oval concrete pool (8.6 x 6.3m, water depth 1.3m) as part of a systematic study on harbour porpoise acoustic signals (Verboom & Kastelein, 1995, 1997, 2003). Recordings were made using a hydrophone (Bruel and Kjaer 8101) suspended in the centre of the pool at mid-water depth, connected to an amplifier (TPD) and a high-speed tape recorder (Racal Store 4D) (Verboom & Kastelein, 1995). The recorded signals were digitised at a sampling frequency of 352,800 Hz at 16-bit. Recorded vocalisations contained a mix of porpoise echolocation behaviour, including echolocation clicks and buzzes. Summary details on each of the recordings used during the playback experiment are outlined in Table 1. General observation clicks, i.e. regular spatial-orientation echolocation clicks, were recorded from a free-swimming porpoise scanning its environment. Due to this scanning behaviour, echolocation clicks recorded predominantly consisted of off-axis echolocation, i.e. porpoise clicks emitted away from the centre of the echolocation beam, hence not at the maximum power of a directional echolocation beam. Object investigation buzzes, i.e. high repetition signals used to identify target objects (and often used in highly discriminatory tasks such as foraging or social behaviour), were recorded from a stationary porpoise at the centre beam (i.e. on-axis, targeted echolocation).

Table 1: Summary details of the recorded harbour porpoise vocalisations. General observation refer to a free-swimming porpoise scanning the environment; Object investigation refers to targeted high repetition signals used to identify target objects.

	Duration (sec)	Recorded echolocation	Activity	Porpoise	Peak frequency	Associated literature for more information	Calculated source levels (re 1 µPa @ 1 m)
**Rec1**	0.55	10 clicks + buzz	General observation	adult	126 kHz	(Verboom, 2022a)	125 dB
**Rec2**	2.8	HF buzz	Object investigation	young	140 kHz	(Verboom, 2022b)	125 dB
**Rec3**	2.3	2x buzz	Object investigation	adult	116 kHz	(Verboom, 2023a)	120 dB
**Rec4**	2.25	3x buzz + clicks	General observation	adult	116 kHz	(Verboom, 2023b)	123 dB
**Rec5**	0.72	6 double clicks	General observation	adult	132 kHz	(Verboom, 2023c)	132 dB

Most of the clicks recorded contained both high-frequency and low-frequency components (Verboom, 2023c). Harbour porpoise adapt their source level according to their environment (Dähne *et al.,* 2020) and circumstances (i.e. when performing certain tasks). Maximum porpoise source levels are expected to be around 175 dBrms or around 190 dBpp re 1 µPa/1m in open-water conditions. As the recording of the playback vocalisations took place in a small pool, it was not possible to measure the emitted levels due to the acoustic reflections within

the pool. In addition, click properties and pulse shape may have differed from recordings conducted in free-field, or open-water conditions.

Playback experiment

Playbacks of harbour porpoise vocalisations were transmitted at 100m increments from the PAM mooring, starting at 100m, and ranging up to a maximum of 600m from the moored static PAM devices, resulting in six playback stations per transect. Two transects were conducted at different bearings to the moored devices, and each transect had two replicates (Figure 1). Transect one took place on a bearing of 295° North at the farthest playback station, and the heading was maintained for the duration of the transect, although some slight variation was recorded due to the drift of the boat. Transect 2 was conducted at a bearing of 8.5° North. The distance of each playback station to the moored static PAM devices was determined using the boat's GPS, calculating a linear distance from the marked device position and GPS waypoint of the boat. GPS waypoints were recorded for each playback station to ensure that the exact locations could be revisited for the replication of the transect. Replicates of transect lines 1 and 2 were completed following the same trajectory, directly after the completion of the respective transect to increase the comparability of replicates. Playbacks along transect 1 were conducted in the morning, whereas those conducted along transect 2 were carried out in the afternoon. GPS waypoints for the moored PAM devices were recorded at the start of both transect 1 and transect 2 to account for any change in positioning throughout the day due to tidal state and currents.

Figure 1: Map showing the locations of the two transect lines conducted for the playback experiment, with playback stations at increments of 100m to 600m from the PAM devices indicated by individual points. Grey box indicates the position of the moored PAM devices. Note that the moored devices were in slightly different latitudinal/longitudinal positions between the morning and afternoon due to slight movement due to currents and tide, the distance between the two points was 35 m. Map insert shows the location of the experimental site in the context of Ireland.

Recorded harbour porpoise vocalisations were transmitted using a computer running PAMGUARD connected to a National Instruments Ltd 6361 usb-box and amplified by a VP2000 amplifier. Porpoise signals were transmitted with an output gain setting of 30dB and 50dB at each playback station, played through an omnidirectional piezo-electric transducer (TC4033: Teledyne Reson A/S, Denmark, with a transmitting sensitivity of 137 dB re 1 µPa/V for 130 kHz signal) suspended from the boat at a depth of circa 2m below the water surface. At each playback station, recordings (n= 5) were played sequentially on repeat for one minute at 30dB, and 50dB amplification. Each individual playback is defined as the distinct transmission of each recording, per each gain setting, at each station, resulting in a total of 10 distinct playbacks per station.

In addition to the playback experiment, a SoundTrap placed 1m from the transducer, at the same depth, was used to monitor the playback output Sound Pressure Level (SPL). Source levels are expressed in dB relevant to a reference unit and can be defined as SPL at 1m from the source (Au & Hastings, 2008; Erbe & Thomas, 2022). SoundTrap data were exported via the SoundTrap Host software, and .wav file was imported to Audacity where SPL was extracted using the *Measure SPL* plugin. The SoundTrap was factory-calibrated with a receiving sensitivity of 176.3 dB re 1µPa/V. Sensitivity is provided for both high and low gain settings and represents the SPL for a normalised (±1.0) wav file with a full-scale signal i.e. 2.0 units peak to peak.

Unlike traditional hydrophone systems, SoundTraps integrate the recorder and hydrophone, resulting in a fixed relationship between sound pressure and the resultant wav file data, eliminating the need for voltage calculations. Audacity reports SPL in units of dB re full scale. To scale the SoundTrap data, we followed methods outlined in the SoundTrap user guide (Ocean Instruments, 2021), where the SPL value (dB re 1 µPa) equals the Audacity SPL reading, plus end-to-end calibration (176.3 dB in this case) minus 3dB. One SPL measurement was taken for each recording across the full spectrum, though variation may exist in different spectrum components. Source levels were calculated between 120 and 132 dB re 1 µPa @ 1 m (Table 1), with some variation due to differing peak frequencies

Data analysis

Data from the deployed C-POD and F-POD were exported using FPOD.exe Version 1.1 (Chelonia Ltd, 2022). To assess which playbacks were detected by each static PAM device, the raw click files (CP1 & FP1) files were initially inspected. Field data forms were consulted to identify the timestamps of each of the playbacks, and it was noted whether a valid detection was identified within this period. This resulted in binary data as detected (1), or not detected (0). CP1 and FP1 files were further processed by Chelonia's KERNO train classifier to identify if any of the playbacks were identified as a click train, either in the correct species class of NBHF (Narrowband high frequency i.e. harbour porpoise in this case) or as a click train but with unclassified species class. The resulting CP3 and FP3 files were subsequently inspected, similar to the raw files, and any instances where a click train was identified during playback was recorded as detected (1).

SoundTrap data were exported via the SoundTrap Host software, and .wav files were imported to Audacity for visual inspection of the spectrograms during the playback period (see S1 Appendix for inspection criteria). As with the data analysed from the C-POD and F-POD, a playback was noted as detected (1) or not detected (0) for the periods corresponding to each playback.

Detection function model fitting

Based on distance sampling theory, the probability of detection can be described as a function of distance to the PAM device (Buckland *et al.,* 2001). The detection function was modelled for each static PAM device using a binary Generalised Additive Model (GAM), with "detected" (1) or "not detected" (0) as the binary response variable following similar methods outlined in Nuuttila *et al.* (2018) and Amundin *et al.* (2022). Distance of the playback from the devices was included as an explanatory variable along with a suite of additional variables that may influence the detection of a playback. These included transect number, source level, water depth (range 18 - 40m), and tide (as a continuous variable between 0-3 representative of the rule of twelfths, based on a smooth rate of tidal flow reaching a maximum velocity halfway between low and high tide). All numerical variables were modelled as thin-plate regression splines, limited to a maximum of 5 degrees of freedom to avoid overly complicated models.

Transect number was included in the model as a factor. Stepwise model selection was performed where non-significant interactions were dropped from the model (starting with the least significant) and model validation was repeated. Models were compared using AIC to choose the best and final model.

The GAM was implemented using the *gam* function in the *mgcv* Package (Wood, 2017), with a binomial error structure and logit link function. Variance and 95% confidence intervals (CIs) were calculated using a nonparametric bootstrap. This approach was used to estimate the detection function g(x) (Buckland *et al.,* 2001), i.e. the probability of detecting a porpoise vocalisation given it is at distance x from the device. The fitted detection functions were then used to estimate the Effective Detection Radius (EDR) for each static acoustic device (Kyhn *et al.,* 2012), using Equation 1:

Equation 1: $$\hat{P} = \frac{2}{w^2}\int_0^{w} x\hat{g}(x)dx$$

$\hat{P}$ is computed as the average probability of detecting a porpoise vocalisation within distance w of the detector. Based on standard distance sampling applications, this assumes uniform detections over the space surveyed by the detectors (i.e. within some horizontal distance w (truncation distance), at which detection probability is assumed to be 0). Here we used a truncation distance of 500m based on previous studies (Amundin *et al.,* 2022), and then integrated out distance *x*.

The EDR ($\hat{p}$) was calculated from the estimated probability (P) using the following equation:

Equation 2: $$\hat{p} = \sqrt{\hat{P}}w^2$$

A similar methodology was also applied by (Nuuttila *et al.,* 2018; Amundin *et al.,* 2022) to compute the Effective Detection Area (EDA) of static acoustic monitoring devices as a

prerequisite for density estimation. EDA is defined as a horizontal circle around the logger where as many detections are missed as are detected outside the circle during a 1-second period (based on distance sampling frameworks e.g., Buckland et al., 2001). This is calculated in 2 dimensions rather than 3 dimensions as animal density is calculated per unit area and not volume. Variation in EDA due to differences in depth can be to some extent captured by including depth as a covariate in analyses (Amundin et al., 2022). Although no density estimation was carried out in the current study, to calculate EDA ($\mathscr{v}$), EDR was converted to EDA, using Equation 3, for each of the devices to investigate comparability with previous studies (division by 1,000,000 is to produce a result in km^2):

$$\text{Equation 3:} \quad \mathscr{v} = \frac{\pi * \hat{p}^2}{1{,}000{,}000}$$

As described above, EDA is defined in 2 dimensions although PAM is typically omnidirectional, represented by a sphere in 3 dimensions. Consequently, for comparison we also calculated the detection range of the devices in a spherical representation, defined as Effective Detection Volume (EDV). EDV represents the theoretical volume in which PAM can detect porpoise vocalisations, assuming open water and ignoring the influence of bottom or surface (e.g. reflections or sediment absorption), also assuming an omnidirectional sensor (Directivity Index (DI) = 0 dB). Note, actual detection capabilities will be influenced by the DI of the hydrophone used and PAM detections are only recorded when the porpoise transmitting beam (approx. +/- 16 degrees around the primary axis) is directed towards the hydrophone.

$$\text{Equation 4: } EDV = \frac{\frac{4}{3} * \pi * \hat{p}^3}{1{,}000{,}000{,}000}$$

These analyses were repeated for each static PAM device. They were also used to investigate the effect that porpoise vocalisation type (i.e. spatial orientation echolocation clicks hereafter referred to as clicks, or buzzes) may have on the EDR of playbacks. As the recorded vocalisations used for the playbacks represented a variety of porpoise echolocation behaviour, a subset of the data was used. Recording 3 was chosen to represent a “buzz only”

playback, while recording 5 was chosen to represent a “click only” playback. In the GAM’s used to investigate the effect of vocalisation type, distance and recording number (3 or 5) were included as the explanatory variables, while all other model components remained consistent with the previous analysis.

Results

There were no cetacean sightings throughout the duration of the playback experiment. Across the two transects, 240 playbacks of harbour porpoise vocalisations were conducted. Of these playbacks, 100 were detected by the SoundTrap, 90 were detected by the F-POD, and 63 were detected by the C-POD (Table 2). Of the playbacks detected by the C-POD, 34 were recognised as click trains by the Chelonia KERNO classifier with 12 classified as NBHF trains. The KERNO-F classifier for the F-POD identified 18 of the playback sequences as click trains, while only 2 were classified as NBHF origin. No playbacks were detected on any of the static acoustic monitoring devices beyond the 500m stations (Table 2). The SoundTrap and the F-POD both recorded maximum detection distances below the 500m range, while the C-POD had maximum detection of the playbacks within 300m of the recording devices.

Table 2: Number of detected playbacks per distance (out of a maximum of 40) for each of the devices. Numbers in parentheses for F-POD and C-POD indicate the number of playbacks that were recognised as click trains by the KERNO classifier, with the later representing those that were recognised to be NBHF, the rest remaining unclassified).

**Distance of playback station to PAM devices (m)**	**SoundTrap**	**F-POD**	**C-POD**
**600**	0	0	0
**500**	0	0	0
**400**	4	1	0
**300**	25	13	1
**200**	32	36 (5,1)	22 (7)
**100**	39	40 (13, 1)	40 (27, 12)
**Total (240)**	100	90	63

GAMs explained 68.4-81.1% of the deviance across the static PAM devices (Table 3). There was a significant increase in the detection of playbacks on all devices with decreasing distance (Table 3). For the SoundTrap and C-POD, in addition to distance, there was an increase in detections of playbacks with an increased source level. The detection of playbacks by the C-POD was also significantly influenced by transect, with a greater number of detected playbacks from transect 1. Tidal flow was not found to be significant in any of the models and was omitted during the model selection process.

Table 3: Final model summaries including deviance explained (Dev. Expl.) for GAMs investigating the factors effecting the detection of playbacks of harbour porpoise vocalisations for all PAM devices. Significant interactions are indicated by *p*-value <0.05, and bold text.

	**SoundTrap model (Dev. Expl. – 68.4%)**		**F-POD model (Dev. Expl. -75%)**		**C-POD model (Dev. Expl. - 81.1%)**	
	p-value	Effect size	p-value	Effect size	p-value	Effect size
**Distance**	**<0.001**	38.48	**<0.001**	30.13	**<0.001**	17.38
**Depth**	0.058	3.58	0.104	7.82	-	-
**Source level**	**<0.001**	3.85	-	-	0.003	3.00
**Transect**	0.101	1.64	-	-	**0.009**	2.62
**Tidal flow**	-	-	-	-	-	-

The probability of detection of the playback of harbour porpoise vocalisations was estimated for each PAM device, along with 95% bootstrap confidence intervals (S2 Appendix). Based on this detection probability, estimated EDR was predicted for all static acoustic monitoring devices (Table 4). The calculated EDR for detecting the playbacks of harbour porpoise vocalisations under the same conditions was the greatest for the SoundTrap, estimated at 297m (95% CI: 239-363) (Figure 2). The EDR for the F-POD was 241m (95% CI: 211 - 282) and the EDR for the C-POD was the lowest at 220m (95% CI: 202 - 242) (Figure 2). The fitted detection function model was also used to predict an EDA per each of the devices (Table 4).

An EDA of 0.276 km^2 was calculated for the SoundTrap, 0.181 km^2 for the F-POD, and 0.153 km^2 for the C-POD (Figure 2). Using Effective Detection Volume (EDV) to represent the detection of harbour porpoise vocalisations in three dimensions shows that the omnidirectional detection range of the F-POD is 54% that of the SoundTrap, while the C-POD's range is 41% comparatively (Table 4).

Table 4: Calculated Effective Detection Radii (EDR) and Effective Detection Area for the PAM devices, and EDR for the playback of distinct vocalisation types.

**SAM Device**	**All Data Analysis**			**Distinct vocalisation types analysis**	
	EDR	EDA	EDV	Vocalisation type	EDR
**SoundTrap**	297m (95% CI: 239-363).	0.276 km^2	0.11 km^3	Click only	319m (95% CI: 254 - 475)
				Buzz only	246m (95% CI: 168 - 406)
**F-POD**	241m (95% CI: 211-282)	0.181 km^2	0.059 km^3	Click only	296m (95% CI: 248 - 350)
				Buzz only	263m (95% CI: 216 - 317)
**C-POD**	220m (95% CI: 202-242)	0.153 km^2	0.045 km^3	Click only	235m (95% CI: 195 - 291)
				Buzz only	204m (95% CI: 161 - 253)

Figure 2: Detection ranges of SoundTrap, F-POD and C-POD shown as Effective Detection Radius (EDR), and Effective Detection Area (EDA) based on the detection probability of harbour porpoise vocalisation playbacks.

A subset of the data was used to investigate the effect of playbacks of different vocalisation types, i.e. click only, or buzz-only vocalisations. These models showed a significant difference between detection of clicks and buzzes for the SoundTrap, but not for either the F-POD or C-POD. Lower EDR was estimated for the playback of vocalisations consisting solely of echolocation buzzes, however, it can be noted that CIs are large in some cases reducing the reliability of estimates (Table 4). The EDR for buzz playback was 73m lower than the click playback for the SoundTrap. A difference of 31-33m in EDR was found for the C-POD & F-POD despite the relationship not being significant in the models.

Discussion

Long-term passive acoustic studies are often integral to monitoring cetacean occurrence, behaviour, abundance, and habitat preference and subsequent changes therein. Choosing the appropriate devices when establishing a monitoring programme is key, as comparative studies have shown that static acoustic devices may vary in their detection rates even when deployed simultaneously (Sarnocinska *et al.,* 2016; Jacobson *et al.,* 2017b; Todd *et al.,* 2023). Differing detection capabilities between devices can make it difficult to compare studies, or relative abundance between areas using different devices. Such discrepancies have been shown between C-PODs and full bandwidth SoundTraps (Sarnocinska *et al.,* 2016; Jacobson *et al.,* 2017b), and between C-PODs and their F-POD successor (Todd *et al.,* 2023). There is a clear need to quantify the differences in detection rates as a function of detection ranges. This study addressed this fundamental knowledge gap by comparing the detection ranges of three commonly used PAM devices: C-POD, F-POD and SoundTrap. While the maximum detection ranges of the SoundTrap and the F-POD were found to be similar (400 - 500m), and both greater than that of the C-POD, the SoundTrap detected the greatest number of playbacks overall, highlighting the greater precision of continuous recording hydrophones. Results also showed that there were differences in the detection ranges based on vocalisation

type, with the detection range of buzzes consistently less than that of clicks across all PAM devices. The differences in detection range between devices emphasizes the needed for careful consideration when choosing PAM devices for monitoring. This is particularly important when monitoring protected areas or offshore developments, it is vital that species or groups are sampled as consistently as possible over time to detect genuine changes in occurrence or density and ensure important areas are afforded appropriate protections.

The estimated EDA for the SoundTrap (0.279 km^2) was the greatest of all three static PAM devices and more than double the estimated EDA of the C-POD (0.153 km^2). The detection range of the F-POD (0.181 km^2) was also found to be greater than the C-POD EDA for this site, although less than the mean C-POD EDA (0.219 km^2) reported from the Baltic region (Amundin *et al.,* 2022). However, it should be noted that considerable variation was found in the EDA across different months and monitoring stations throughout the Baltic study (range: 0.034 km^2- 0.742 km^2), reflecting different environmental conditions with different soundscapes across the region. Our results show that the detection probability of porpoise playbacks under the same environmental conditions and ambient soundscape, varied between devices. No playbacks were detected on any of the moored devices beyond 500m, in line with the maximum detection range of wild porpoise echolocation assumed by Sveegaard *et al*. (2011). However, it should be emphasized that the EDR depends on the source level of the playback device, so the present study may have shown EDR above 500m when higher SLs would have been used.

Despite the SoundTrap and F-POD having the same maximum detection distances, the estimated EDR of the SoundTrap was considerably greater, 297m versus 241m respectfully, reflecting the greater number of playbacks at the 300m and 400m stations detected by the SoundTrap. It was also estimated that the omnidirectional detection range of the F-POD, in terms of EDV, was 54% of the SoundTrap, while C-POD was estimated at 41%, highlighting large differences between the SoundTrap and the click detectors. Greater consistency in detection of playbacks on the SoundTrap may reflect differences in device sensitivities and

critical bandwidth, or the SoundTrap's enhanced ability to detect echolocation clicks from broadband recordings compared to click detectors such as C-POD/F-POD. The click detectors in-built detection algorithms likely also influence how the playbacks, and subsequently in-situ detections are recorded by the devices compared to full spectrum hydrophones such as the SoundTrap. For the C-POD, our EDR of 220m is comparable with previous research by Nuuttila *et al.* (2018) who reported a mean EDR of 188m for harbour porpoise, but it should be noted that our estimate was for one device, and EDR has been shown to vary considerably between C-POD devices. A recent study derived an estimated detection range for SoundTrap and F-POD at a maximum of 105m through tracking wild echolocating porpoises with drones (Cosentino et al., 2023). This range is notably less than the detection ranges derived in the current study, potentially highlighting that wild porpoise detection ranges may be considerably lower than experimental ranges observed through playback experiments (for instance due to ambient noise or other local conditions).

Furthermore, it is wise to assume that variability between individual PAM devices also exists. However, while some variation between the same device type could be attributed to differences in sensitivity, these should have a relatively minor effect if the devices are correctly and regularly calibrated (Dähne *et al.,* 2013). The greater detection ranges found in this study for the SoundTrap and F-POD in comparison to the C-POD could, at least in part, explain previous research showing increased detection rates for harbour porpoise from the SoundTrap (Sarnocinska *et al.,* 2016; Jacobson *et al.,* 2017b), and the F-POD (Todd *et al.,* 2023). While advances in technology resulting in increased detection rates and improved detection ranges is positive, it is important to consider what consequences this may have for comparison with historical data, given that these may underestimate occurrence and abundance compared to newer devices. Interpreting data from newer devices may also be confounded, as declines in species abundance could potentially be masked by the increased detection rates of fewer individuals. This highlights that comparing data derived from multiple devices is a very nuanced matter that should be given careful consideration.

For the C-POD only, there was a significant difference in the detection of the playbacks between the two transects. As the effect of transect was not shown for the other devices, it is unlikely that time of day (with transect two conducted in the afternoon as opposed to the morning), or orientation of device in water (which would influence co-deployed devices) are key drivers of this difference. One plausible explanation for the decreased detection probability for transect two is the potential influence of environmental noise, which may be greater for transect two due to its position closer to land and in slightly shallower water. C-PODs have been reported to be highly sensitive to environmental noise levels (Clausen *et al.,* 2019) within the 20-160 kHz noise band. This may mask porpoise detections and can cause C-PODs to 'max out', limiting further porpoise detections. Studies on F-PODs have shown that environmental noise has less influence on detection rates (Todd *et al.,* 2023), and do not 'max out' under high ambient noise conditions. It is therefore important to consider the environment and ambient noise levels when choosing deployment sites for PAM equipment.

The EDR of buzzes was found to be lower than that of clicks for all devices, although, the difference in EDR calculated for the SoundTrap was twofold higher than that of the other devices. Other studies on cetaceans have shown that buzzes can typically have lower sound output levels than clicks, with differences of up to 20dB reported for sperm whales (*Physeter macrocephalus*) (Madsen *et al.,* 2002), beaked whales (Blainville's) (Madsen *et al.,* 2005), and 10dB for harbour porpoise (DeRuiter *et al.,* 2009). In the current study, calculated source levels for the "buzz only" playback were 8dB lower than those of the "click only". Therefore, the reduced source levels will be the key influence on the reduced estimated EDR across the devices rather than a difference in the detection ability for the different vocalisations. Buzzes are a good indication of porpoise foraging behaviour (Verfuß *et al.,* 2009; Miller, 2010), and can help identify critical foraging habitat. However, if buzzes are consistently detected at reduced distances from static PAM devices than clicks, due to reduced source levels, foraging behaviour derived from PAM devices is potentially underestimated due to the reduced search area. It is important to note that wild harbour porpoise echolocation behaviour is highly directional (Au *et al.,* 1999; Macaulay *et al.,* 2020), and for clicks, this is partly compensated by head-scanning behaviour to ensonify a greater area (Verfuß *et al.,* 2009). However, for buzzes, echolocation is targeted towards an object, e.g. prey, in a close-range focused beam.

Given an omnidirectional transducer was used to conduct this playback experiment, detection ranges of wild porpoise (for all vocalisation types) are potentially less than those estimated across the acoustic devices due to increased directionality of their echolocation.

In conclusion, this study found significant differences in detection ranges across three commonly used static PAM devices. We provide EDR and EDA for these devices under the same ambient soundscape. As C-PODs reach the end of their operational lives and studies adopt or integrate F-PODs or SoundTraps into acoustic monitoring programmes, increased detection ranges are an undoubted benefit, but consideration needs to be given to the comparability with older devices in time-series analysis. These findings highlight the importance of considering device-specific detection ranges for accurately estimating density and abundance, as well as for designing effective monitoring strategies, particularly for sensitive species. If feasible, cetacean monitoring projects using static PAM devices should determine and report the EDR/EDA to aid comparability of detection rates or densities across sites and studies.

Acknowledgements: This work was funded by the Irish Research Council Government of Ireland Postgraduate Scholarship Scheme (Project ID: GOIPG/2019/2173). We would like to thank Nick Tregenza for the loan of the F- POD that supported this project and the technical support provided by the Chelonia team. All those who provided discussions during the conception of this study including Ollie Boisseau, Mats Amundin, Hanna Nuuttila, Jamie MacAulay, Magnus Wahlberg and Luke Rendell. We would also like to thank Sam Cox for providing the SoundTrap for this experiment. We are grateful to Michael Collins skipper of the Kestrel, and for various field assistants for facilitating the fieldwork in Roaringwater Bay. Open access funding provided by IReL.

Data Availability Statement: All processed data and R code files to reproduce the results given in this paper are available in the following repository https://github.com/Ntodd95/playbacks

References:

Amundin, M., Carlström, J., Thomas, L., Carlén, I., Koblitz, J., Teilmann, J., Tougaard, J., Tregenza, N., Wennerberg, D., Loisa, O., Brundiers, K., 2022. Estimating the abundance of the critically endangered Baltic Proper harbour porpoise (*Phocoena phocoena*) population using passive acoustic monitoring. *Ecology and Evolution*, *12(2),* p.e8554.

Au, W.W. and Hastings, M.C., 2008. *Principles of marine bioacoustics* (Vol. 510). New York: Springer.

Barlow, J., Griffiths, E.T., Klinck, H., Harris, D.V., 2018. Diving behavior of Cuvier's beaked whales inferred from three-dimensional acoustic localization and tracking using a nested array of drifting hydrophone recorders. *The Journal of the Acoustical Society of America*, *144(4),* pp.2030-2041.

Barlow, J., Cheeseman, T., Trickey, J.S., 2021a. Acoustic detections of beaked whales, narrow-band high-frequency pulses and other odontocete cetaceans in the Southern Ocean using an autonomous towed hydrophone recorder. *Deep Sea Research Part II: Topical Studies in Oceanography*, *193*, p.104973.

Barlow, J., Fregosi, S., Thomas, L., Harris, D., Griffiths, E.T., 2021b. Acoustic detection range and population density of Cuvier's beaked whales estimated from near-surface hydrophones. *The Journal of the Acoustical Society of America*, *149(1),* pp.111-125.

Blumstein, D.T., Mennill, D.J., Clemins, P., Girod, L., Yao, K., Patricelli, G., Deppe, J.L., Krakauer, A.H., Clark, C., Cortopassi, K.A., Hanser, S.F., 2011. Acoustic monitoring in terrestrial environments using microphone arrays: applications, technological considerations and prospectus. *Journal of Applied Ecology, 48(3)*, pp.758-767.

Bouffaut, L., Taweesintananon, K., Kriesell, H.J., Rørstadbotnen, R.A., Potter, J.R., Landrø, M., Johansen, S.E., Brenne, J.K., Haukanes, A., Schjelderup, O., Storvik, F., 2022. Eavesdropping at the speed of light: Distributed acoustic sensing of baleen whales in the Arctic. *Frontiers in Marine Science, 9*, p.901348.

Buckland, S.T., Anderson, D.R., Burnham, K.P., Laake, J.L., Borchers, D.L., Thomas, L., 2001. *Introduction to distance sampling: estimating abundance of biological populations.* Oxford University Press.

Carlström, J., 2005. Diel variation in echolocation behavior of wild harbor porpoises. *Marine Mammal Science, 21(1),* pp.1-12.

Cauchy, P., Heywood, K.J., Merchant, N.D., Risch, D., Queste, B.Y., Testor, P., 2023. Gliders for passive acoustic monitoring of the oceanic environment. *Frontiers in Remote Sensing, 4,* p.1106533.

Chelonia Ltd (2022) www.chelonia.co.uk.

Clausen, K.T., Tougaard, J., Carstensen, J., Delefosse, M., Teilmann, J., 2019. Noise affects porpoise click detections–the magnitude of the effect depends on logger type and detection filter settings. *Bioacoustics, 28(5),* pp.443-458.

Cosentino, M., Tougaard, J., Dähne, M. & Kyhn, L. A. 2023. Detection function using simultaneous drone videos and acoustic recordings. Aarhus University, DCE – Danish Centre for Environment and Energy, 18 pp. Scientific Report No. 566

Dähne, M., Verfuß, U.K., Brandecker, A., Siebert, U., Benke, H., 2013. Methodology and results of calibration of tonal click detectors for small odontocetes (C-PODs). The *Journal of the Acoustical Society of America, 134(3),* pp.2514-2522.

Dähne, M., Bär, T., Gallus, A., Benke, H., Herold, E., Stilz, P., 2020. No need to shout? Harbor porpoises (*Phocoena phocoena*) echolocate quietly in confined murky waters of the Wadden Sea. *The Journal of the Acoustical Society of America, 148(4),* pp.EL382-EL387.

Davis, R. W., Kiszka, J., Harcourt, R., Meekan, M., Bejder, L., Rodriguez, D. H., Stockin, K.A., 2022. Small Cetacean Conservation: Current Challenges and Opportunities, Lausanne: Frontiers Media SA

DeRuiter, S.L., Bahr, A., Blanchet, M.A., Hansen, S.F., Kristensen, J.H., Madsen, P.T., Tyack, P.L., Wahlberg, M., 2009. Acoustic behaviour of echolocating porpoises during prey capture. *Journal of Experimental Biology, 212(19)*, pp.3100-3107.

Erbe, C., Thomas, J.A., 2022. *Exploring Animal Behavior Through Sound: Volume 1*: *Methods* (p. 517). Springer Nature.

Harris, D.V., Miksis-Olds, J.L., Vernon, J.A., Thomas, L., 2018. Fin whale density and distribution estimation using acoustic bearings derived from sparse arrays. The *Journal of the Acoustical Society of America, 143(5),* pp.2980-2993.

Jacobson, E.K., Forney, K.A., Barlow, J., 2017. Using paired visual and passive acoustic surveys to estimate passive acoustic detection parameters for harbor porpoise abundance estimates, *The Journal of the Acoustical Society of America, 141(1),* pp. 219–230.

Jacobson, E.K., Merkens, K.P., Forney, K.A., Barlow, J., 2017b. Comparison of harbor porpoise (*Phocoena phocoena*) echolocation clicks recorded simultaneously on two passive acoustic monitoring instruments. Available at: https://eirenjacobson.info/JacobsonEtAl_NOAATM_2017.pdf.

Kyhn, L.A., Tougaard, J., Thomas, L., Duve, L.R., Stenback, J., Amundin, M., Desportes, G., Teilmann, J., 2012. From echolocation clicks to animal density—Acoustic sampling of harbor porpoises with static dataloggers. *The Journal of the Acoustical Society of America, 131(1),* pp.550-560.

Macaulay, J.D., Malinka, C.E., Gillespie, D., Madsen, P.T., 2020. High resolution three-dimensional beam radiation pattern of harbour porpoise clicks with implications for passive acoustic monitoring. *The Journal of the Acoustical Society of America, 147(6),* pp.4175-4188.

Madsen, P., Wahlberg, M., Møhl, B., 2002. Male sperm whale (*Physeter macrocephalus*) acoustics in a high-latitude habitat: implications for echolocation and communication. *Behavioral Ecology and Sociobiology, 53*, pp.31-41.

Madsen, P.T., Johnson, M., De Soto, N.A., Zimmer, W.M.X., Tyack, P., 2005. Biosonar performance of foraging beaked whales (*Mesoplodon densirostris*). Journal of *Experimental Biology, 208(2),* pp.181-194.

Malinka, C.E., Atkins, J., Johnson, M.P., Tønnesen, P., Dunn, C.A., Claridge, D.E., de Soto, N.A., Madsen, P.T., 2020. An autonomous hydrophone array to study the acoustic ecology

of deep-water toothed whales. *Deep Sea Research Part I: Oceanographic Research Papers, 158,* p.103233.

Marques, T.A., Thomas, L., Ward, J., DiMarzio, N., Tyack, P.L., 2009. Estimating cetacean population density using fixed passive acoustic sensors: An example with Blainville's beaked whales. *The Journal of the Acoustical Society of America, 125(4),* pp.1982-1994.

Marques, T.A., Thomas, L., Martin, S.W., Mellinger, D.K., Ward, J.A., Moretti, D.J., Harris, D., Tyack, P.L., 2013. Estimating animal population density using passive acoustics. *Biological reviews, 88(2),* pp.287-309.

Miller, L.A., 2010. Prey capture by harbour porpoises (*Phocoena phocoena*): A comparison between echolocators in the field and in captivity, *Journal of the Marine Acoustics Society, 37(3),* pp. 156–168.

National Parks & Wildlife Service. 2014. Site synopsis: Roaringwater Bay and islands SAC site code: 000101. Available at: https://www.npws.ie/sites/ default/files/ protected-sites/ synopsis/SY000 101.pdf.

Nuuttila, H.K., Brundiers, K., Dähne, M., Koblitz, J.C., Thomas, L., Courtene-Jones, W., Evans, P.G., Turner, J.R., Bennell, J.D., Hiddink, J.G., 2018. Estimating effective detection area of static passive acoustic data loggers from playback experiments with cetacean vocalisations. *Methods in Ecology and Evolution, 9(12),* pp.2362-2371.

Ocean Instruments, 2021. SoundTrap ST300 and ST4300 series user guide. September 2021. Available from: https://www.oceaninstruments.co.nz/wp-content/uploads/2015/04/ST-User-Guide.pdf

Sarnocinska, J., Tougaard, J., Johnson, M., Madsen, P.T., Wahlberg, M., 2016, July. Comparing the performance of C-PODs and SoundTrap/PAMGUARD in detecting the acoustic activity of harbor porpoises (*Phocoena phocoena*). *In Proceedings of meetings on acoustics (Vol. 27, No. 1*). AIP Publishing.

Schaffeld, T., Bräger, S., Gallus, A., Dähne, M., Krügel, K., Herrmann, A., Jabbusch, M., Ruf, T., Verfuß, U.K., Benke, H., Koblitz, J.C., 2016. Diel and seasonal patterns in acoustic

presence and foraging behaviour of free-ranging harbour porpoises. *Marine Ecology Progress Series, 547,* pp.257-272.

Sveegaard, S., Teilmann, J., Berggren, P., Mouritsen, K.N., Gillespie, D., Tougaard, J., 2011. Acoustic surveys confirm the high-density areas of harbour porpoises found by satellite tracking. *ICES Journal of Marine Science, 68(5),* pp.929-936.

Todd, N.R.E., Kavanagh, A.S., Rogan, E., Jessopp, M.J., 2023. What the F-POD? Comparing the F-POD and C-POD for monitoring of harbor porpoise (*Phocoena phocoena*). *Ecology and Evolution, 13(6),* p.e10186.

Todd, N.R.E., Jessopp, M., Rogan, E., Kavanagh, A.S., 2022. Extracting foraging behavior from passive acoustic monitoring data to better understand harbor porpoise (*Phocoena phocoena*) foraging habitat use. *Marine Mammal Science, 38(4),* pp.1623-1642.

Todd, N.R.E., Cronin, M., Luck, C., Bennison, A., Jessopp, M., Kavanagh, A.S., 2020. Using passive acoustic monitoring to investigate the occurrence of cetaceans in a protected marine area in northwest Ireland. *Estuarine, Coastal and Shelf Science, 232,* p.106509.

Van Parijs, S.M., Clark, C.W., Sousa-Lima, R.S., Parks, S.E., Rankin, S., Risch, D., Van Opzeeland, I.C., 2009. Management and research applications of real-time and archival passive acoustic sensors over varying temporal and spatial scales. *Marine Ecology Progress Series, 395,* pp.21-36.

Verboom, W.C. (2022a) Analysis of Harbour porpoise (*Phocoena phocoena*) click series (1). MEM2022015.

Verboom, W.C. (2022b) Analysis of Harbour porpoise (*Phocoena phocoena*) click series (2). MEM2022016.

Verboom, W.C. (2023a) Analysis of Harbour porpoise *(Phocoena phocoena*) click series (3). MEM2022017.

Verboom, W.C. (2023b) Analysis of Harbour porpoise (*Phocoena phocoena*) click series (4). MEM202301.

Verboom, W.C. (2023c) Low-frequency component in Harbour porpoise (*Phocoena phocoena*) clicks. MEM202302.

Verboom, W.C. and R.A. Kastelein. 2003. Structure of harbour porpoise (*Phocoena phocoena*) acoustic signals with high repetition rates. *In: Echolocation in bats and dolphins (Eds. J.A. Thomas, C. Moss and M. Vater)* University of Chicago Press, Chicago, USA, 40-43.

Verboom, W.C., Kastelein, R.A., Read, A.J., 1997. Structure of harbour porpoise (*Phocoena phocoena*) click train signals. *The biology of the harbour porpoise. De Spil Publishers, Woerden*, pp.343-362.

Verboom, W.C., Kastelein, R.A., 1995. Acoustic signals by harbour porpoises (*Phocoena phocoena*). *Harbour porpoises: Laboratory studies to reduce bycatch*, pp.1-39.

Verfuß, U.K., Miller, L.A., Pilz, P.K. Schnitzler, H.U., 2009. Echolocation by two foraging harbour porpoises (*Phocoena phocoena*). *Journal of Experimental Biology, 212(6),* pp.823-834.

Zein, B., Woelfing, B., Dähne, M., Schaffeld, T., Ludwig, S., Rye, J.H., Baltzer, J., Ruser, A., Siebert, U., 2019. Time and tide: Seasonal, diel and tidal rhythms in Wadden Sea Harbour porpoises (*Phocoena phocoena*). *Plos one, 14(3),* p.e0213348.

Zimmer, W.M., 2011. *Passive acoustic monitoring of cetaceans*. Cambridge University Press.

Zimmer, W.M., Harwood, J., Tyack, P.L., Johnson, M.P., Madsen, P.T., 2008. Passive acoustic detection of deep-diving beaked whales. *The Journal of the Acoustical Society of America, 124(5),* pp.2823-2832.

figure 1

Click here to access/download;Figure;Figure 1 Map and insert300.tiff

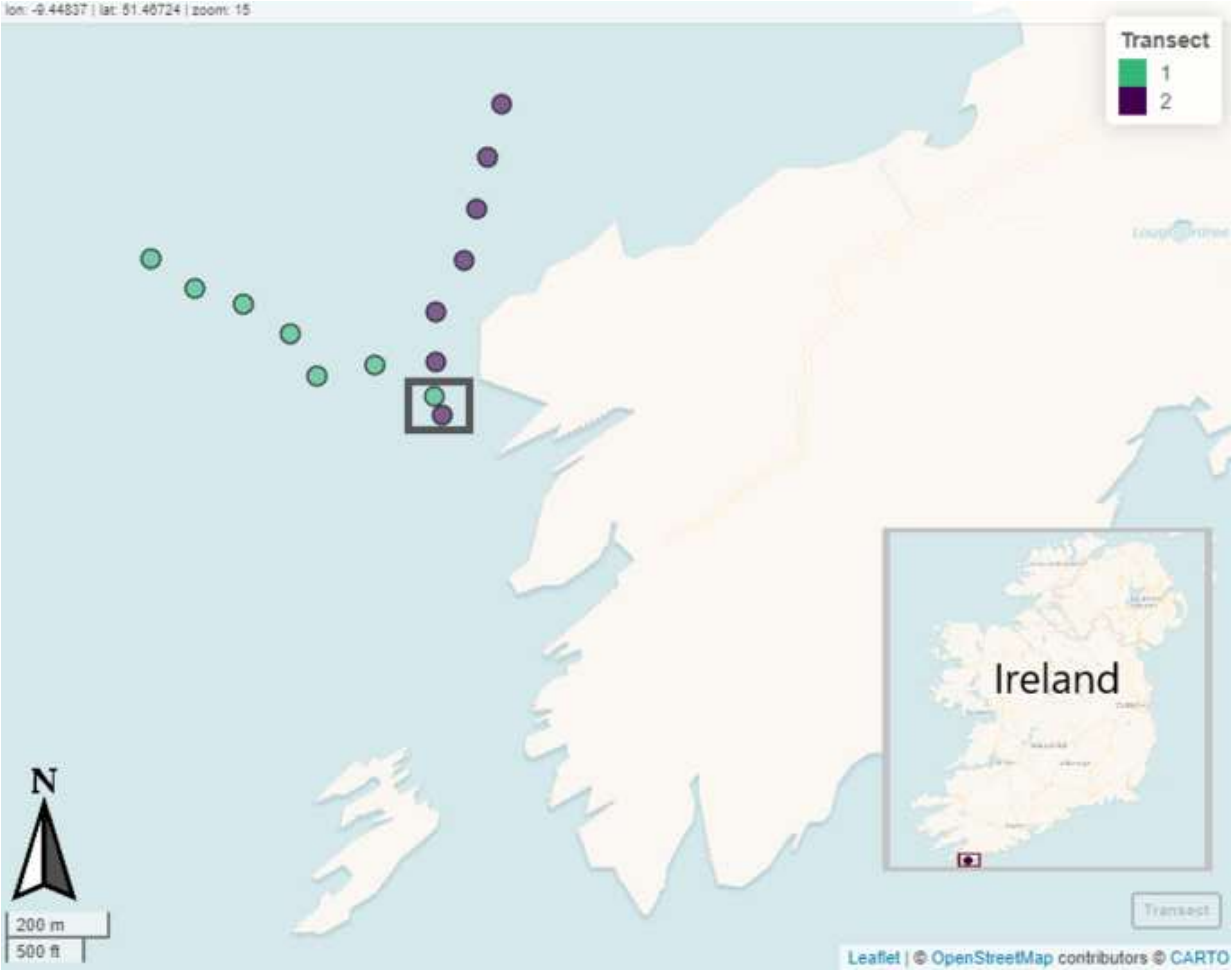

figure 2

Click here to access/download;Figure;Figure 2_300.tiff

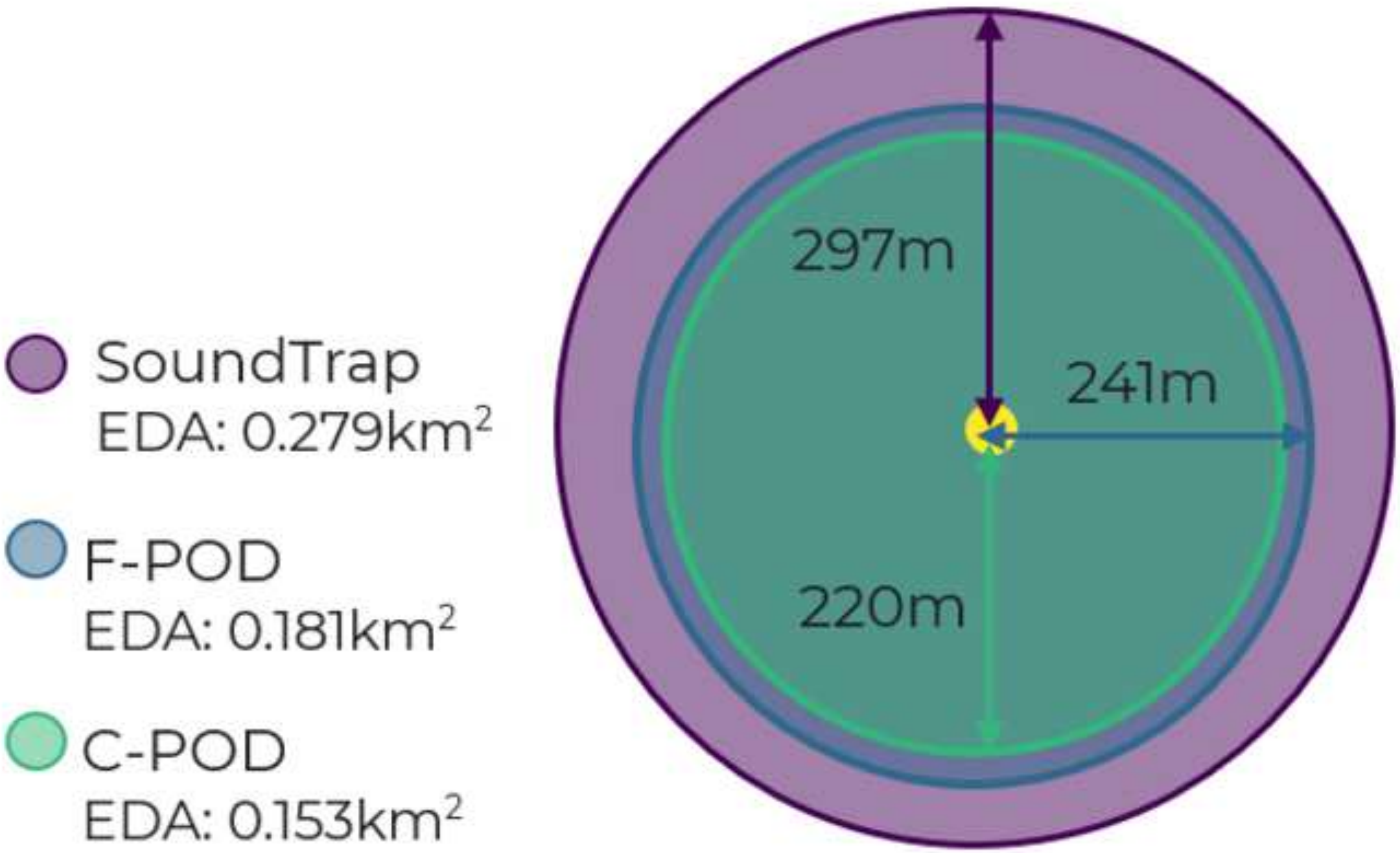

Appendix 1

Click here to access/download
Supporting Information
S1 Appendix_submit.docx

Appendix 2

Click here to access/download
Supporting Information
S2 Appendix_submit.docx
