# Supplementary material for: Can you hear me? Playback experiment highlights detection range differences between commonly used PAM devices: C-POD, F-POD and SoundTrap
Source: PLoS One. 2025 Apr 9;20(4):e0320925. doi: 10.1371/journal.pone.0320925 (PMC11981142; doi:10.1371/journal.pone.0320925)
Supplement: Attachment — Submitted filename: PONE-D-24-43959 Response to Reviewers.docx [file pone.0320925.s005.docx]

Dear Dr Paiva,

**Re. Manuscript** **PONE-D-24-43959**

Many thanks for taking the time to provide editorial and reviewer comments on our manuscript “Can you hear me? Playback experiment highlights porpoise detection range differences between commonly used PAM devices: C-POD, F-POD, and SoundTrap” and inviting a revised version. We would like to thank the reviewers for their positive and constructive comments and have outlined below how we have addressed them, with responses below each comment. We hope you agree that the manuscript is much improved and now meets the standard for publication in PLOS ONE.

Journal requirements:

When submitting your revision, we need you to address these additional requirements.

1. Please ensure that your manuscript meets PLOS ONE's style requirements, including those for file naming. The PLOS ONE style templates can be found at <https://journals.plos.org/plosone/s/file?id=wjVg/PLOSOne_formatting_sample_main_body.pdf> and <https://journals.plos.org/plosone/s/file?id=ba62/PLOSOne_formatting_sample_title_authors_affiliations.pdf>"

*Response: Style requirements have been reviewed and amendments made accordingly*

2. In your Methods section, please provide additional information regarding the permits you obtained for the work. Please ensure you have included the full name of the authority that approved the field site access and, if no permits were required, a brief statement explaining why

*Response: No permits were required and the following sentence has been added to the manuscript.* “Following consultation with the National Parks and Wildlife Service it was established that no permits or additional permissions were required to conduct the research, however the following measures were implemented to reduce potential disturbance to wild cetaceans.”

3.  Thank you for stating the following financial disclosure:

“This work was funded by the Irish Research Council Government of Ireland Postgraduate Scholarship Scheme (Project ID: GOIPG/2019/2173).”

Please state what role the funders took in the study.  If the funders had no role, please state: ""The funders had no role in study design, data collection and analysis, decision to publish, or preparation of the manuscript.""

If this statement is not correct you must amend it as needed.

Please include this amended Role of Funder statement in your cover letter; we will change the online submission form on your behalf.

*Response: Funding statement amended, please see cover letter revision for updated statement.*

4.  Thank you for stating the following in the Acknowledgments Section of your manuscript:

“This work was funded by the Irish Research Council Government of Ireland Postgraduate Scholarship Scheme (Project ID: GOIPG/2019/2173). We would like to thank Nick Tregenza for the loan of the F- POD that supported this project and the technical support provided by the Chelonia team. All those who provided discussions during the conception of this study including Ollie Boisseau, Mats Amundin, Hanna Nuuttila, Jamie MacAulay, Magnus Wahlberg and Luke Rendell. We would also like to thank Sam Cox for providing the SoundTrap for this experiment. We are grateful to Michael Collins skipper of the Kestrel, and for various field assistants for facilitating the fieldwork in Roaringwater Bay. Open access funding provided by IReL.”

We note that you have provided funding information that is not currently declared in your Funding Statement. However, funding information should not appear in the Acknowledgments section or other areas of your manuscript. We will only publish funding information present in the Funding Statement section of the online submission form.

Please remove any funding-related text from the manuscript and let us know how you would like to update your Funding Statement. Currently, your Funding Statement reads as follows:

“This work was funded by the Irish Research Council Government of Ireland Postgraduate Scholarship Scheme (Project ID: GOIPG/2019/2173).”

Please include your amended statements within your cover letter; we will change the online submission form on your behalf.

*Response: Funding information removed from Acknowledgements as requested and funding statement has been amended.*

5.  Please include captions for your Supporting Information files at the end of your manuscript, and update any in-text citations to match accordingly. Please see our Supporting Information guidelines for more information: <http://journals.plos.org/plosone/s/supporting-information>.

*Response: Supporting information captions added as per the guidelines*

Reviewer 1

Reviewer #1: This is a useful article especially to researchers conducting long-term monitoring using different devices, with good insights on EDR/EDA implications. As I mentioned, the duration of the recording itself (or recording type) could be a factor in the reception of the signal by the PAM device, and therefore should be investigated if it is a relevant factor. It is well-written, and the structure is clear and concise, but I suggest improving the figures and adding the figure from the supplementary to the article (see my comment in the pdf). I would also like to see more details on the results of model selection.

*Response:* We thank the reviewer for their positive feedback and helpful comments on our manuscript. We have taken the feedback on board, improving the figures and adding additional information on the models (particularly related to AIC). In relation to the duration of the recordings, all playbacks were transmitted as a 1-minute playback loop. Because the calls were standardised across all stations, any differences between devices are explored by including variables such as source level and type of vocalisations in further analysis. We have also provided a breakdown of detections by call type in Table 2 that shows all calls were detected with almost 100% success by all devices, at least at short distances, so recording duration in this case is unlikely to be a factor in terms of differences in detectability between devices. Please see specific responses to the detailed comments below.

Detailed comments:

Line 25: Determined the

*Response: Amended as suggested*

Line 45-47: insert reference

*Response: Reference added*

Line 48-49: in parenthesis

*Response: Amended as suggested*

Line 90: suggest replacing “and” with “while

*Response: Amended as suggested*

Line 98: “as much as” these words can be removed

*Response: Amended as suggested*

Line 112: I'm missing the information on when the deployment and playbacks were conducted, which is important for literature comparison. Water temperature, and therefore season, is also an important factor in the detection of sound (sound speed is affected by temperature).

*Response: We agree the context of season and water temperature is important, so a sentence has been added to note when the experiment took place and the water temperature- “*The playback experiment was conducted on 02/04/2023 with a water temperature of 10°C at the deployment site.”

Line 117: would be nice to add a figure of the sensor setup next to the map

*Response: Figure of senor setup has been added next to the map*

Line 183: “Inset map”

*Response: Amended as suggested*

Line 219: word “files” doubled

*Response: Duplicate word deleted*

Line 241: Duration of the playback could also affect detection. Perhaps it should be added as a variable?

*Response: Duration of playback was standardised across all experiments, with each playback lasting a total of one minute at each playback station. Therefore this was not considered as a variable as it did not vary between playbacks.*

Line 270: delete “by”

*Response: Amended as suggested*

Line 291: Since for EDV, it assumes an omnidirectional sensor, this sentence only applies to calculations of EDA/EDR?

*Response: For clarity, we have moved this sentence and added direct reference to EDR/EDA.*

Line 300: I can see now that recording number is included, which in a way includes duration (but also vocalization type and other factors). But maybe also interesting to see duration as a separate explanatory variable..

*Response: All playbacks were transmitted for a total of one minute duration, there should be no difference between the recordings in terms of duration so we did not consider this as an explanatory variable.*

Line 300: And 3 or 5 in parentheses are not clear to me

*Response: 3 or 5 was deleted as was potentially misleading and it is described in the previous sentence which recordings are used in the subset*

Line 315: classify each detection by recording type in this table. is the reception of the device influenced by the recording type (and not necessarily the vocalisation type as you mentioned)?

*Response:* As suggested, we have classified each detection by recording in the table. This demonstrates that all recordings were reliably detected by all the devices at a close range and there is no single recording that is systematically under recorded compared to the others. See Line 306 for the amended table.

Line 315: Latter

*Response: Amended as suggested*

Line 315: Remove Parenthesis

*Response: Amended as suggested*

Line 316: ?

*Response: It looks like the line number ended up in the table, not sure how that happened but it seems to have resolved itself in the revised manuscript.*

Line 320: why not for the f-pod? was this in the discussion or i missed it?

*Response: This factor was not retained in the F-POD model. Implication of this added to discussion, see comment below.*

Line 324: I'm missing the AIC values for each model selection.. should include this in the supplementary

*Response: Added AIC tables to supplementary -S2 File.*

Line 324: affecting

*Response: Amended as suggested*

Line 324: do these dashes mean that the factor was excluded in the model selection? state this in the caption. what does it imply that the source level is not relevant for the final model for f-pod?

*Response: Amended as suggested in caption. Sentence regarding the potential implication for the F-POD added to the discussion, see line 421.*

Line 324: this is also significant

*Response: Amended as suggested*

Line 327: This is an important figure. Should be included in the paper and not as a supplementary. And better to combine all 3 graphs into one.

*Response: We tried combining all 3 graphs into one and it was difficult to differentiate between the devices due to the overlapping 95% CI. As such, for clarity, we have kept the 3 separate graphs. This figure has been removed from supplementary and has been included in the paper. See Fig 2.*

*Graph amended. All three detection function graphs combined and added to main text*

Line 333: “per device”

*Response: Amended as suggested*

Line 335: “Using”- “the”, “to represent"- “which represents

*Response: Amended as suggested*

Line 349: the word lower gives an impression that this is about vertical distance rather than horizontal. It is implied by EDR, but to make it easier to read I suggest using the word "smaller" or lesser"

*Response: Amended as suggested*

Line 349: insert EDR "the EDR for"

*Response: Amended as suggested*

Line 356: “device” not devices

*Response: Amended as suggested*

Line 371: emphasize not emphasizes, need not needed

*Response: Amended as suggested*

Line 373: Remove comma and replace with “.” Or separate into two sentences

*Response: Amended as suggested*

Line 375: Protection not protections

*Response: Amended as suggested*

Line 377: Important to mention the exact month/season of sampling for comparison

*Response: Exact month has now been mentioned within the methods and results for comparison and context.*

Line 390: “if” not when

*Response: Amended as suggested*

Line 392: “respectively” not respectfully

*Response: Amended as suggested*

Line 395-397: insert Table no.

*Response: Amended as suggested.*

Line 405-406: which study? has this only been reported for c-pods and not the other PAM devices?

*Response: Reference added to Nuuttila et al.*

Line 424: could be potentially

*Response: Amended as suggested*

Line 428: insert p-value, or table no.

*Response: Amended as suggested.*

Line 471: “sensitive species” is unclear

*Response: Clarity added, amended to “species of concern for conservation and management”*

Line 472: not just report, but also normalize/weight abundances/estimates using EDR/EDA

*Response: This is a good point! Amended* “If feasible, cetacean monitoring projects using static PAM devices should determine the EDR/EDA to aid comparability of detection rates and to normalise density and abundance estimates across sites and studies”

Figure 2: Figure can be improved, circles are not aligned.. important to mention the date/time of playback experiments used to calculate these values in the caption

*Response: Thank you for pointing that out. Figure has been aligned properly and date of the experiment has been added to the figure caption.*

Reviewer #2: Dear Authors

Thank you for conducting the research and preparing this manuscript comparing the detection performance of commonly used acoustic recorders. I found the manuscript to be well written, concise, and interesting. I think this manuscript will be of great interest to the audience of this journal. I don't have a lot comments except for the few in the marked up document.

*Response:* We thank the reviewer for taking the time to review this manuscript and for their positive reception. Please see responses to specific comments below.

Detailed comments:

Key words: Maybe replace these words on the title with words that are not on the title for easier indexing.

*Response: Amended as suggested*

Line 203: Shouldn't this value be negative? Hydrophone sensitivities are usually " negative because the amount of voltage produced by a sound is less than 1 volt per 1 pascal, the standard underwater pressure used for measurement".

*Response: Yes, thanks for noticing this omission. Amended as suggested.*

Line 275: Write number name for numbers below 10., Write out name in full.

*Response: Amended as suggested*

Line 378: Double of 0.153 is 0.306, so 0.279 is less than 0.306. I think what you can say here is "almost double".

*Response: Amended as suggested*

Line 430: What about changes in water temperature over time? Did you look at this?

*Response: The experiment was conducted over a matter of hours therefore in relatively stable conditions, no changes in water temperature where found over this time period. However, as per comments of Reviewer 2, the date the experiment was conducted, as well as water temperature have been added to the manuscript, as water temperature is indeed an important factor that influences sound propagation, so it is important to have this context.*

Line 443: “Two-fold higher”- I don't think the difference is two-fold based on Table 4, can you please recheck this?

*Response: I checked Table 4 and this is correct. The difference between the EDR for C/FPOD was 31-33m, compared to 73m for the soundtrap. I’ve reworded the sentence slightly for clarity.* “The EDR of buzzes was lower than that of clicks for all devices. However, the difference in EDR for the SoundTrap was twice as large as the difference observed for the other devices”
