# Supplementary material for: Can you hear me? Playback experiment highlights detection range differences between commonly used PAM devices: C-POD, F-POD and SoundTrap
Source: PLoS One. 2025 Apr 9;20(4):e0320925. doi: 10.1371/journal.pone.0320925 (PMC11981142; doi:10.1371/journal.pone.0320925)
Supplement: Attachment — Submitted filename: PONE-D-24-43959(1) Response to Reviewers.docx [file pone.0320925.s006.docx]

Dear Dr Paiva,

**Re. Manuscript** **PONE-D-24-43959(1)**

Thank you for your oversight on our manuscript, "Can you hear me? Playback experiment highlights detection range differences between commonly used PAM devices: C-POD, F-POD, and SoundTrap." We appreciate the reviewers' positive and constructive feedback. Below, we outline how we have addressed their comments, with specific responses provided for each. We believe these revisions have significantly improved the manuscript and that it now meets the publication standards for PLOS ONE.

Review Comments to the Author

**Reviewer #2:** Thank you for the revised manuscript. My comments and suggestions were properly addressed, and I have no further comments.

**Reviewer #3:** General comments

I think this is a very useful experiment and paper but it could use more detail and clarification. I realize this is not the first set of review comments and in the detailed comments below I have made an effort to be explicit about what information would be helpful. An additional sentence or two in the identified paragraphs should provide the necessary information. If the results from one of the gain settings were excluded, this needs to be explained and the total number of playbacks included in the analysis should be consistent. If the results from both settings were used, I would suggest explaining why they were combined in the results.

The rationale behind and composition of the playback methods, especially where the method differs from those in the cited methods papers, should be justified. For example, Nuuttila et al. (2018) reported EDR results for both artificial and recorded harbour porpoise calls and by detected versus classified click trains (Figure 4). The selection of the five recordings is not explained. A previous reviewer questioned the duration of the recordings, and from the manuscript it is not clear how the 0.55 - 2.8 sec clips were combined into a one minute playback, how many times the clips were played, or at what gain. I understand a bit better after reviewing the playback2.2.csv file on your Github, but this information needs to be in the manuscript. A diagram of how the playbacks were composed would help the reader and even a condensed version of the results spreadsheet could be included in an appendix.

I would like more information to understand how your analysis supports the comparison of EDR for the recorded buzzes versus clicks. My understanding is that you have calculated the source level of the playback but are assuming the difference in these values could represent the difference in true source levels. However, this seems like a coincidence as in theory the buzz could have been detected at a higher received level than the click train (in the pool) depending on the distance and orientation of the captive harbour porpoise from the receiver. You reference the 8dB difference in the discussion, but I would recommend briefly explaining your rationale and assumptions in the methods. The other differences between the recordings (duration, number of clicks, frequency) and how this might impact detection range should also be discussed.

It should be clear throughout the manuscript that you have estimated detected range of the playbacks and not of harbour porpoise, given the source levels of the playbacks are much lower than the maximum source levels referenced. The title of the manuscript might imply that the values reported represent harbour porpoise detection ranges, but the emphasis should be on comparing the devices.

I would suggest you review the use of the word “their” (when referencing the instruments) and the term “vocalisations” throughout the manuscript. I realize this is a common practice, but as clicks are not produced using vocal cords, some odontocete scientists prefer to use “clicks”, “acoustic signals” or “calls”.

*Response:* *Thank you for your insightful feedback and helpful comments, which have led to significant improvements in the manuscript. In response to your general comments, we have revised the methods to clearly outline the playback procedure, including the sequence of recordings, gain settings, and total number of playbacks at each station. We have justified the selection for the five recordings used in our playback experiment, emphasizing their representative characteristics of echolocation behaviours. Additionally, we clarified that the comparison of EDR for clicks versus buzzes is based on distinct call types rather than assumptions about differences in source levels. The manuscript now consistently reflects that we estimated the detection range of the playbacks, and the title has been modified accordingly. We also reviewed and amended the use of “their” and “vocalisations” where appropriate. We trust that these revisions address your comments and enhance the clarity and robustness of our findings. Please see below for our specific responses to your detailed comments.*

Detailed comments:

Line 54-55: beaked whale researchers are transitioning from “Cuvier’s” to “goose-beaked”

*Response: Amended as suggested*

Line 65: change “their successor” to “its successor”

*Response: Amended as suggested*

Lines 92-96: Does the literature suggest why FPODs and SoundTraps detected more than C-PODs other than ambient noise? Here or in the methods would be a good place to provide a bit more information about how the click train detectors work on C-PODs and F-PODs as this likely has a significant impact on what would be detected in a long term monitoring study.

*Response: There is still uncertainty about this matter in the literature* *I have added a sentence clarifying that the C-POD’s zero-crossing detection algorithm may be more influenced by ambient noise conditions compared to other detectors. See line 94.*

Line 97: change “...their detection ranges…” to “its detection range” or “the detection range”

*Response: Amended as suggested*

Line 100-101: would suggest a less broad statement such as “assessing the effect of porpoise call type including echolocation clicks and foraging buzzes” and I still recommend that more information is needed about why the recordings selected can be representative of these two types of echolocation.

*Response: Amended as suggested. More information on why the recordings were selected is provided later in the text, see line 334.*

Line 114: What frequency was the high pass filter set to?

*Response: This is a predefined setting within the ‘Audio options’ of the SoundTrap software which can be turned on or off. Clarity has been added to the text, see line 121.*

Line 131-148: An explanation of how / why the five recordings were selected would be helpful, and why artificial signals were not used. You could also explain how / why Rec3 and Rec5 are representative of the two echolocation types.

*Response: The selected recordings were chosen based on their representativeness of harbour porpoise echolocation signals. Artificial signals were not used because producing such signals was outside the scope of the available resources for this study. This information has been incorporated into the revised text, see lines 144-147.*

*Recordings 3 and 5 were selected for their distinct echolocation behaviours to ensure that the analyses accurately reflected the effects of each call type, as the other recordings contained a mix of both call types. Further clarification has been added to the text. Please see Line 334.*

Line 140-142: Here general observation clicks are defined as being regular echolocation clicks but in Table 1 these recordings include buzzes. I would suggest updating the paragraph or the table for consistency.

*Response: Table one has been updated for clarification.*

Line 144-145: Does off axis echolocation refer to a signal being emitted off centre or the recording of a signal outside of the beam? This sentence could be improved with some rephrasing.

*Response:* *Sentence has been amended to improve clarification that off-axis refers to the emission of the signal from the porpoise.*

Line 155: I assume dBpp refers to peak to peak but it is helpful to state that clearly when it is first referenced as sometimes dBpp is used to denote zero to peak.

*Response: Amended for clarity*

Lines: 154-159: I would recommend briefly addressing these points again in your discussion. (1) The source levels of the playbacks were much lower than maximum porpoise source levels. A brief discussion of how this does or does not impact your results would be helpful. (2) The source levels of the captive porpoise was not calculated. How is the source level of the playback relevant to the comparison of the different click types? (2) Click characteristics from these recordings might be different than those in the wild. Does this impact your results and conclusions? Is there any information about how C-POD and F-POD detectors perform with captive animal signals? It would be helpful to see that you have considered these factors in your interpretations and conclusions.

*Response: These points have been addressed throughout the discussion. 1) For the impact of lower source levels, please see line 436. 2) The source level was not used as an indicator of variation among click types; this distinction has been clarified further in the text and in response to other comments. The relevance of source level is primarily based on literature indicating that buzzes typically have lower source levels than clicks, which aligns with our findings. 3) Discussing the performance of the devices with signals from captive animals is outside the scope of the current study. However, we have added additional discussion recognising that click characteristics of wild porpoise may differ from those in our experiment (see line 511). Given the variety of echolocation behaviours included, we believe the results are representative.*

Line 164-165: It would be helpful if you explained how you decided on the bearings of the two transects.

*Response: Additional information has been provided for clarity, see line 179.*

Line 186: I would suggest replacing “circa” with “approximately”

*Response: Amended as suggested*

Line 188-190: This should be reworded for clarity. It is not clear how the playbacks were composed and it is difficult to follow the numbers referenced here and in the results. In addition, if each recording (n = 5) was played at two gain settings, the use of the gain and how it impacts the results should be clarified. Why were those gain settings selected? Line 190 states there were 10 playbacks per station but in the results (Table 2) it is reported that there were 8 playbacks per recording and 40 playbacks at each station. Was each of the 5 recordings played 4 times at each station (given there were two replicates)? Was the sequence of recordings (Rec1, Rec2, Rec3, Rec4, Rec5, Rec1, Rec2, … etc.) repeated 4 times within one minute? What was the buffer (time gap) between the recordings? How were the two gain values used? From the playback2.2.csv file on your Github it seems that a recording was repeated a certain number of times to make up one minute, and this repeated sequence was played sequentially. This needs to be explained in your methods. It is difficult to evaluate the results from the CPOD or FPOD click detector or the manual review of the sound data in Audacity without understanding how these recordings were compiled and how the playbacks were conducted. A diagram or spectrogram of how the recordings were composed - i.e. what was played at each station - would be helpful.

*Response: Thank you for your detailed comment. We have revised the methods section to clarify the playback sequence, gain settings and total number of playbacks and made reference to the dataset in the repository. See lines 212-227 for the revised text.*

Line 205-208: Given signals were transmitted from a transducer using two different gain settings, I would expect a mean source level or two different estimates of source level. In the playback2.2.csv file on your Github, source levels for playbacks at the 50 dB gain settings are reported. If you excluded the results from one of the gain settings, this needs to be explained - there are 240 rows in your results spreadsheet, if half were excluded the total number of playbacks should add up to 120 (your results report 240 playbacks)? Also, please explain why one SPL measurement was taken across the full spectrum, as often the focus should be on the peak frequencies of interest. Please clarify how you measured and calculated SPL and SL from one or more playbacks of the five different recordings.

*Response:*

*Source levels for both 30 dB and 50 dB gain settings were reported in the dataset, and no results were excluded. The total of 240 playbacks includes both gain settings, with source level included as an explanatory variable in the models. A single SPL measurement was taken across the full spectrum to capture potential frequency variation, particularly where multiple call types were present. SPL values were measured from a test setup at close range prior to the field experiment, following the SoundTrap user guide. I have revised the text for clarity on these matters, see lines 242-247.*

Line 208: Needs a period

*Response: Period added.*

Line 214-215: Can you provide more detail about how you defined a “valid detection”. Nuuttila et al. (2018) provide details that would be helpful here, e.g., they recorded the number of clicks detected and whether only part of the sequence was recorded. They also divided C-POD results based on whether clicks were detected in the raw files, by the click train detector, or as porpoise click trains. Use of results from only raw files should be explained as this is relevant to how well a C-POD or F-POD will perform in long term studies - when the click detector and classification as NBHF will be relied on.

*Response: To define a valid detection, we considered it as the recording of at least one click within the playback period and the appropriate frequency range. We focused on raw detections to capture all potential responses, acknowledging that the click detector requires a minimum of six clicks to classify a click train, which may not reflect weaker detections. We have added additional text for clarity, please see line 255.*

Line 221-225: Similar to my questions above, how was a detection validated? Did the entire one minute recording have to be detected or did partial recordings count?

*Response: A valid detection was defined as recording at least one click within the playback period and appropriate frequency range, meaning partial recordings were included. As per the previous comment we have added clarity on this to the text.*

Line 234: Was the replicate or gain used on the transducer not relevant?

*Response: Gain was not included as this was superseded by including source level and it would not be possible to include both.*

Line 261: Should “wheras” be “wherein” or “within which”?

*Response: Amended as suggested*

Line 263: Missing a “)”

*Response: Amended as suggested*

Line 284-288: The detection of these two recordings, especially on the F-POD and C-POD, could differ due to factors such as frequency, duration, number of clicks, and amplitude. A justification for why those recordings were selected as representative of two echolocation behaviours would be helpful. In addition, if the assumption is that the difference in playback source level is representative of the difference in source level of an echolocation click train versus buzz click train, this needs to be explained and justified by literature.

*Response: Recordings 3 and 5 were selected not for their source levels but for their distinct echolocation behaviours. We have added clarity on this, please see Line 334.*

Line 295: This section and the title of Table 2 contains some information that would be helpful in the methods when explaining how the playbacks were designed and conducted. In the methods (Line 188) states that each playback is the transmission of each recording per gain setting. There is no reference to the different gain settings in the results. This should be clarified in the methods and results. The playback2.2.csv file on your Github shows that the engine as one for the start of your playbacks. Even if this does not impact results, it should be noted. Furthermore, for both tracks 1.1 and 1.2 the measured distance (“true distance” column) from the mooring for stations at 100 and 200 m are recorded as 128.75 m and 225.97 m. This should be explained. Did your include these true distances in your distance analysis or models? If not, you could explain why you assumed it would not alter your results.

*Response:*

*We have revised the Methods section to provide clearer information on how the playbacks were designed and conducted, ensuring alignment with the details in the Results, see line 212.*

*Additionally, we have noted that the engine was briefly on at the beginning of the playback experiment, but this was not expected to impact the results (see lines 126). Regarding the measured distances ("true distance"), slight variations were acknowledged, but we believe these do not alter the results. Further clarification has been added to the text (see line 190).*

Line 300: The F-POD detected 90/240 playbacks, 18 were marked as click trains, and two as NBHF. This represents fairly poor performance by the F-POD; I would expect due to the source level, short duration of the recordings and limited number of clicks. A brief discussion of this and some more details on how the click train detector and NBHF classifier works would be helpful - does it rely on a certain number of clicks to identify a click train? I think it is relevant to know which of the recordings were identified as click trains and which of those were identified as NBHF. This is also why it would be helpful to understand the rationale behind the recordings selected and experiment design. I suggest this is relevant because for long term monitoring I expect the click detector and NBHF classifier would be relied on, as opposed to manual review used to find the 90 detections.

*Response: The performance of the classifiers was not evaluated in great depth to maintain consistency across devices. We have added clarification on the click detector’s operation (see lines 261) and included discussion on the F-POD’s detection of playbacks, classification of click trains/NBHF, and the potential implications for long-term monitoring see (lines 465-469).*

Table 2: Including percentages would be helpful as it is difficult to determine how many playbacks were detected out of the total played for each recording and each station (distance). For example, 100 playbacks were detected by the SoundTrap, which is ~42%. If two gain settings were used, that information should also be in the table.

*Response: Percentages were added to text for overall % detected for each device, see line 347. For clarity, it has been noted that both gain settings were used in the caption of Table 2.*

Line 327: I would suggest including a sentence that reports the EDV values before comparing them as percentages.

*Response: Sentence reporting EDV added as suggested.*

Line 343: Replace the “&” with “and”

*Response: Amended as suggested*

Line 348: suggest replacing “therein” with another word as it’s not the correct use

*Response: Amended as suggested*

Line 350: change “...their detection rates…” to “... detection rates…”

*Response: Amended as suggested*

Line 354: change use of “their”

*Response: Changed to its as per previous suggestion*

Line 359: suggest adding a period after C-POD and making this two sentences

*Response: Amended as suggested*

Line 360: Is “precision” the correct word here?

*Response: Amended for clarity*

Line 361-363: The two recordings used as representative of call types had different source levels, but this has not been linked to the source level of the captive porpoise. If you are assuming that the difference in source levels, as well as other characteristics that impact detection on the devices, is representative of the difference in clicks and buzzes, this needs to be explained. In addition, it should be clear that you have estimated detected range of the playbacks and not of harbour porpoise given the source levels of the playbacks are much lower than the maximum source levels referenced. Given you should have source level and received level values, you could calculate transmission loss and estimate detection range with the theoretical maximum source level.

*Response:* *Differences in source level alone do not fully distinguish clicks from buzzes, as multiple factors influence source levels, but it is likely that source levels is the key factor influencing detectability in this case. We have clarified in the text, line 436. Additionally, we have explicitly stated in the discussion that the estimated detection range applies to playbacks, not harbour porpoise (see lines 410).*

Line 393: “subsequently” means “afterward”, I would suggest selecting a different word here

*Response: Amended as suggested*

Line 400-401: Detection range of wild porpoise could also be greater than experimental estimates depending on environmental conditions, devices, and source levels. You reference this at line 381.

*Response: I have amended the sentence for clarity, see line 456.*

Line 406: Line 397 states that EDR has varied considerably between devices, was this due to a calibration issue?

*Response: This was not clear from the literature unfortunately.*

Line 418: Does this reference the calculated source level of the different recordings or the change in gain used for the transducer?

*Response:* *We have clarified that the variation in source level was influenced by both differences between recordings and the gain settings used (see line 482).*

Line 422: Wouldn’t amplitude of a signal be related to source level?

*Response: Amended for clarity*

Line 425: I’m not sure the difference in bearing between the two transects provides enough information to make conclusions about how the orientation of the devices impacted detection. I assume the devices might rotate or turn while in the water column?

*Response: Devices can turn rotate or turn in the water, but the C-POD and the F-POD were co-deployed in a custom frame, as such would be in the same orientation in the water column. Neither device was found to change orientation in the water column throughout the duration of the experiment.*

Line 442-445: I suggest a brief explanation for why the difference in source level of these two playbacks was assumed representative of the difference between click and buzz source level be provided in the methods. Other than the reduced source level, is it possible the frequency, duration, or number of clicks impacted whether these two recordings were detected? If not, why?

*Response:* *Recordings 3 and 5 were selected not for their source levels but solely for their distinct echolocation behaviours, this has been clarified based on previous comments. The text has been amended to indicate that peak frequency and the number of clicks may influence the detection of these two call types, see line 509.*

Line 453-455: Do you have a reference for this? Directionality of the echolocation beam impacts detection range but is it more an issue of detection probability?

*Response:* *Reference added for the directionality of echolocation.*
